# Participation of OCRL1, and APPL1, in the expression, proteolysis, phosphorylation and endosomal trafficking of megalin: Implications for Lowe Syndrome

**DOI:** 10.3389/fcell.2022.911664

**Published:** 2022-10-20

**Authors:** Lisette Sandoval, Luz M. Fuentealba, María-Paz Marzolo

**Affiliations:** ^1^ Laboratorio de Tráfico Intracelular y Señalización, Departamento de Biología Celular y Molecular, Facultad de Ciencias Biológicas, Pontificia Universidad Católica de Chile, Santiago, Chile; ^2^ Instituto de Ciencias Biomédicas, Facultad de Ciencias de la Salud, Universidad Autónoma de Chile, Santiago, Chile

**Keywords:** megalin, lowe syndrome, ocrl1, APPL1, renal disease, proteolysis, GSK3, insulin

## Abstract

Megalin/LRP2 is the primary multiligand receptor for the re-absorption of low molecular weight proteins in the proximal renal tubule. Its function is significantly dependent on its endosomal trafficking. Megalin recycling from endosomal compartments is altered in an X-linked disease called Lowe Syndrome (LS), caused by mutations in the gene encoding for the phosphatidylinositol 5-phosphatase OCRL1. LS patients show increased low-molecular-weight proteins with reduced levels of megalin ectodomain in the urine and accumulation of the receptor in endosomal compartments of the proximal tubule cells. To gain insight into the deregulation of megalin in the LS condition, we silenced OCRL1 in different cell lines to evaluate megalin expression finding that it is post-transcriptionally regulated. As an indication of megalin proteolysis, we detect the ectodomain of the receptor in the culture media. Remarkably, in OCRL1 silenced cells, megalin ectodomain secretion appeared significantly reduced, according to the observation in the urine of LS patients. Besides, the silencing of APPL1, a Rab5 effector associated with OCRL1 in endocytic vesicles, also reduced the presence of megalin’s ectodomain in the culture media. In both silencing conditions, megalin cell surface levels were significantly decreased. Considering that GSK3ß-mediated megalin phosphorylation reduces receptor recycling, we determined that the endosomal distribution of megalin depends on its phosphorylation status and OCRL1 function. As a physiologic regulator of GSK3ß, we focused on insulin signaling that reduces kinase activity. Accordingly, megalin phosphorylation was significantly reduced by insulin in wild-type cells. Moreover, even though in cells with low activity of OCRL1 the insulin response was reduced, the phosphorylation of megalin was significantly decreased and the receptor at the cell surface increased, suggesting a protective role of insulin in a LS cellular model.

## Introduction

Megalin is an endocytic receptor belonging to the low-density lipoprotein receptors family (Saito et al., 1994; Marzolo and Farfán, 2011). This receptor is found in the apical surface of several epithelia and is highly expressed in the kidney, specifically in the proximal tubular epithelium (PT) (Saito et al., 1994; Farquhar et al., 1995). The function of megalin in epithelial kidney cells is related to the recapture of low molecular weight proteins (LMW) from the glomerular filtrate (Leheste et al., 1999). Megalin ligands, including calcium, albumin, insulin, leptin, parathyroid hormone (PTH), angiotensin II, retinol-binding protein (RBP), and vitamin D binding protein (DBP), are involved in pathological kidney conditions (Marzolo and Farfán, 2011). Renal defects of megalin knockout (KO) mice (Leheste et al., 2003, 1999) include phosphaturia, hypercalciuria, and proteinuria, due to loss of megalin ligands such as RBP, DBP and albumin (Bockenhauer et al., 2008; Bothwell et al., 2011). These defects are explained in terms of inefficient endocytosis of megalin ligands and, importantly, to additional megalin trafficking functions related to the regulation of inorganic sodium phosphate cotransporter (NaPi-IIa) (Bachmann et al., 2004) and sodium-proton exchanger 3 (NHE3) (Alexander and Grinstein, 2009). Also, the lack of megalin is associated to significant ultrastructural changes in the endosomal compartments of PT epithelial cells, including the absence of dense apical tubules, which correspond to the apical recycling endosomes, and other endocytic structures, such as clathrin-coated pits and vesicles (Leheste et al., 1999). On the other hand, under physiological conditions, it is possible to find megalin fragments in the urine (Pisitkun et al., 2004; Thrailkill et al., 2009; Suruda et al., 2017). The predominant species is megalin ectodomain (A-megalin), probably released by shedding, a process that would take place at the cell surface through a mechanism involving the activity of protein kinase C (PKC) and matrix metalloproteases (Zou et al., 2004). Besides, it is also possible to detect, but at low levels, the full-length receptor (C-megalin) present in exosomes (Suruda et al., 2017). C-megalin levels are increased in diabetes patients (Kurita et al., 2022) and negatively correlated with serum 1,25(OH)2D and 24,25(OH)2D (Toi et al., 2019). However, there are still open questions regarding the mechanisms explaining the presence and the change in the levels of megalin in the urine, especially in different pathologies affecting the kidney.

The cytoplasmic domain of megalin has an essential role in determining its apical distribution and recycling (Marzolo et al., 2003; Yuseff et al., 2007; Farfán et al., 2013; Perez Bay et al., 2016). In polarized epithelial cells, internalized megalin follows an endosomal itinerary including the apical sorting endosome (ASE), the common recycling endosome (CRE) (where it meets with basolateral internalized cargoes such as TfR), and the Rab11 positive apical recycling endosome (ARE), from which it recycles to the cell surface (Mattila et al., 2014; Perez Bay et al., 2016; Eshbach and Weisz, 2017). Also, the already mentioned ectodomain shedding can modify megalin surface levels. Besides, it is known that glycogen synthase kinase-3 ß (GSK3ß) binds directly to the megalin cytoplasmic domain (Marzolo and Farfán, 2011) and phosphorylates the PPPSP motif within the intracellular domain of the receptor, decreasing the efficiency of megalin recycling and therefore reducing its cell surface expression (Yuseff et al., 2007).

Lowe Syndrome (LS) is a human pathological and lethal condition caused by mutations in the *OCRL* gene, encoding a phosphatidylinositol 5-phosphatase OCRL1, affecting the brain, eye, and kidney (Lowe et al., 1952; Attree et al., 1992). The disease is characterized by congenital cataracts, central hypotonia, and renal proximal tubular dysfunction (Preston et al., 2020). There are different mutations in *OCRL* causing LS, which decrease the function or expression of the enzyme (Lichter-Konecki et al., 2006; De Matteis et al., 2017). LS patients also exhibit high concentrations of proteins in their urine, including megalin ligands. Besides, the secretion of megalin itself, as A-megalin, seems to be specifically decreased, contrasting with the normal secretion of cubilin, a megalin co-receptor (Norden et al., 2002; Suruda et al., 2017). The mechanism that explains the A-megalin decrease is not known.

OCRL1 modulates the endocytic trafficking of several cargo receptors (Choudhury et al., 2005; Erdmann et al., 2007; Noakes et al., 2011; Vicinanza et al., 2011). We have described significant alterations in endocytic trafficking of various receptors, including megalin, in OCRL1 silenced cells (Vicinanza et al., 2011). This last study showed defects in early endosomal compartments characterized by the abnormal presence of phosphatidylinositol 4,5-P2, a preferential substrate of OCRL1 (Zhang et al., 1998), and by the ectopic accumulation of actin filaments (Dambournet et al., 2011; Vicinanza et al., 2011; Kühbacher et al., 2012) that impede efficient recycling of cargoes. As a consequence of these defects, cells deficient in OCRL1 exhibit a reduction in the cell surface expression of megalin. Besides, data obtained from humanized Lowe Syndrome animals (Festa et al., 2019) and zebrafish (Oltrabella et al., 2015) indicate that cell surface and total megalin are decreased in proximal tubule cells. On the other hand, APPL1 is an endocytic protein that associates with OCRL1 (Erdmann et al., 2007). In some LS OCRL1 mutants, the interaction with APPL1 is disrupted (McCrea et al., 2008; Noakes et al., 2011). The presence of megalin in APPL1 endosomes is also reduced in OCRL1 knock-down cells (Vicinanza et al., 2011).

Here we explored the expression of megalin in a “cellular Lowe condition”, finding a post-transcriptional regulation of megalin levels in OCRL1 silenced cells. We also evaluated megalin proteolysis and ectodomain secretion. When OCRL1 levels were low, the secretion of the receptor ectodomain was decreased. These results indicate that OCRL1 knock-down (KD) cells show a phenotype that mimics the situation found in the urine of LS patients (Norden et al., 2002; Suruda et al., 2017). In addition, we found that silencing of APPL1 also reduced cell surface expression and secretion of the megalin ectodomain to cell culture.

The reported reduction and surface expression of megalin in LS could be partly explained by defects in endosomal recycling due to ectopic actin polymerization in the endosomes as described (Vicinanza et al., 2011) but also can be the result of changes in intracellular signaling processes that increases GSK3ß activity. Our results showed that the basal megalin phosphorylation was rather decreased in LS conditions and that the endosomal distribution of the receptor depended on its phosphorylation status as well as on the activity of OCRL1. Interestingly, we uncovered a role of insulin in megalin phosphorylation and cell surface expression, finding that the stimulation of cells with the hormone reduces megalin phosphorylation, consistent with a decreased activity of GSK3ß, and increases its surface expression. Finally, although insulin signaling was decreased in cells with reduced function of OCRL1, megalin was still less phosphorylated and more present at the plasma membrane, indicating that although less efficient, insulin could promote megalin recycling in LS conditions.

## Materials and methods

### Antibodies and reagents

The monoclonal anti-HA and the polyclonal anti-human cytoplasmic domain of megalin antibodies have been described before (Marzolo et al., 2003; Yuseff et al., 2007). Polyclonal antibody against human OCRL was described before (Vicinanza et al., 2011). Two rabbit anti-APPL1 human antibodies were used, one kindly provided by Dr. David Kaplan (Lin et al., 2006), and the other was from Cell signaling (D83H4; XP^®^ Rabbit mAb #3858). Anti-tubulin monoclonal antibody (MAB3408)was purchased from Chemicon (Temecula, CA, USA). Antibodies against E-cadherin and GSK3ß (610202) were from BD Bioscience-Pharmingen (San Jose, CA, USA). The antibodies for phospho-GSK3ß (Ser-9) (D85E12), phospho-AKT S473 (D9E) 4060, AKT 9272, EEA1 (C45B10), Rab7 (D95F2) were purchased from Cell signaling. Alexa Fluor-594 goat anti-rabbit IgG, Alexa Fluor-488 goat anti-mouse IgG, and anti-HA-Alexa-488 were purchased from Molecular Probes (Europe BV, Leiden, the Netherlands). LiCl and Coomassie blue were obtained from Winkler (Santiago, Chile). Protein A-agarose and G-agarose were from Pierce (Rockford, IL, USA) [^35^S] methionine/cysteine was obtained from ICN (Costa Mesa, CA, USA) [^32^P] orthophosphate was purchased from the Chilean Commission of Nuclear Energy (CCHEN, Chile). All tissue culture media, serum and plastics, were from Life Technologies, Inc. (Rockville, MI, USA). Matrix metalloproteinase inhibitor (GM6001) was from Calbiochem (San Diego, CA, USA). YU142670 was obtained from Merck & Co., Inc. (Kenilworth, NJ, USA). Sulfo-NHS-LC-biotin and Immunopure Streptavidin-agarose were from Pierce (Rockford, IL, USA).

### Plasmids and primers sequences

Megalin mini receptor (mMeg) was generated from a human kidney cDNA library (Marzolo et al., 2003; Farfán et al., 2013). Plasmids for phosphomimetic mMeg (mMeg S170D), non-phosphorylatable mMeg (mMeg S170A) and mMeg lacking the ectodomain (Meg0) were described previously (Yuseff et al., 2007; Marzolo and Farfán, 2011). mCherry-Rab11 was kindly provided by Dr. Alexis Gautreau (Derivery et al., 2009). Human OCRL1-EGFP was described before (Vicinanza et al., 2011). Plasmids for short hairpin RNAs were MISSION^®^ pLKO.1-puro Non-Target shRNA Control Plasmid DNA (Sigma-Aldrich), pLKO vectors purchased from Open Biosystems (shOCRL1 5′- GCC​AAG​TAT​AAG​AAA​GTT​CAA -3′ and shAPPL1 5′- GCA​TTG​TTA​GAA​CCT​CTA​CTT-3′). The primers used in quantitative PCR reactions were as follows: megalin forward, 5′- CTG​CTC​TTG​TAG​ACC​TGG​GTT​C -' 3; megalin reverse, 5′- TCG​GCA​CAG​CTA​CAC​TCA​TAA​C -3; glyceraldehyde-3-phosphate dehydrogenase forward, 5′- TCA​AGG​CTG​AGA​ATG​GGA​AG -`3; glyceraldehyde-3-phosphate dehydrogenase reverse, 5′- AGC​AGA​AGG​GGC​AGA​GAT​G -`3.

### Cell lines and culture conditions

LLC-PK1 epithelial cells, derived from porcine kidney were cultured in alpha-MEM supplemented with 7.5% FBS containing 100 U/ml penicillin and 100 mg/ml streptomycin) and 2 mM glutamine (Invitrogen). These cells have been previously used in studies on proximal tubule function and megalin expression (Marzolo et al., 2003; Yuseff et al., 2007; Cabezas et al., 2019, 2011). MDCK epithelial cells are derived from dog kidneys and correspond to the distal tubule. These cells have been used previously to study megalin and LRP1 trafficking (Marzolo et al., 2003; Yuseff et al., 2007; Farfán et al., 2013) and were obtained from ATCC. HEK 293T cells were used to produce lentivirus as described (Sotelo et al., 2014). HeLa cells were maintained in DMEM with 10% FBS, 2 mM glutamine, and antibiotics. All the cells were grown at 37°C in humidified air containing 5% CO2.

### Lentivirus production and cell transfection

Plasmids encoding shRNA, pCMVR8.91, and VSVg were transfected in HEK293 cells using calcium method phosphate (Chen and Okayama, 1987). The supernatant with lentivirus was collected after 48 h and used to infect cells in the presence of 8 μg/ml of polybrene. Stably silenced clones were selected with 2 μg/ml puromycin in growing media for 3 days after infection. Cells were transfected with Lipofectamine 2,000 (Invitrogen), according to the manufacturer’s instructions. For the generation of stably-expressing minireceptors, mMeg or Meg0 (Marzolo et al., 2003), cells lines were transfected and selected using 0.8 mg/ml of G418 for 2 weeks and then maintained with 0.4 mg/ml of G418.

### Western blot

Cells were lysed with lysis buffer (PBS containing 1% Triton X-100, 1 mM glycerophosphate, 1 mM sodium orthovanadate, 5 mM sodium fluoride, and the protease inhibitors 2 mM PMSF, 1 mM pepstatin, 2 μM antipain, 1 μM leupeptin, and 0.3 μM aprotinin). Extracts were centrifuged at 12.000 rpm for 10 min, and the pellet was discarded. Protein from lysates and the immune complexes (for immunoprecipitation samples) were boiled in Laemmli sample buffer (62.5 mM Tris-HCl, pH 6.8; 2% w/v SDS, 10% v/v glycerol, and 5% ß-mercaptoethanol) and then analyzed by SDS-PAGE or tris-tricine under reducing conditions. Gels were transferred to an Immobilon-P membrane (Millipore, Billerica, MA). The membranes were blocked in TBS containing 1% Triton X-100 and 3% BSA and subjected to incubation overnight with primary antibodies and for 2 h with secondary antibodies. Blots were developed with the ECL system from Amersham Life Science (Arlington Heights, IL, USA) and analyzed with ImageJ.

### Quantitative PCR

Total RNA was isolated using RNA-Solv (Omega Bio-Tek, Norcross, GA, USA) following the manufacturer’s instructions and treated with DNAse I. Then, the reverse transcription was performed with random primers and RevertAidTM MMuLV Reverse Transcriptase in the presence of RNAseOUT (Fermentas Glen Burnie, MD, USA). PCR reactions were performed using a 7,500 Real-Time PCR System (Applied Biosystems, Carlsbad, CA, USA) and Brilliant SybrGreen I (Stratagene). Results were analyzed with the 7,500 System Software.

### Metabolic labeling

To measure megalin biosynthesis, the cells were incubated with 200 uCi/ml of [35S]-methionine/cysteine at 37°C for 4 h, followed by a wash with ice-cold PBS and lysing procedure. Radiolabeled mMeg was immunoprecipitated with rabbit polyclonal anti-megalin antibody (Marzolo et al., 2003) and protein A–agarose beads (Pierce). Samples were separated by SDS-PAGE and visualized by autoradiography.

### Megalin half-life and intracellular proteolytic products accumulation

For mMeg half-life measurement, 5 × 10^6^ cells/cm2 (shControl and shOCRL LLC-PK1) were plated on 6-well plates and treated with cycloheximide (100 μM) for indicated times in culture media (DMEM) without serum, up to 12 h. At the end of the incubation, cells were lysed using lysis buffer (PBS containing 1% Triton X-100, 1 mM glycerophosphate, 1 mM sodium orthovanadate, 5 mM sodium fluoride, and the protease inhibitors 2 mM PMSF, 1 mM pepstatin, 2 μM antipain, 1 μM leupeptin, and 0.3 μM aprotinin). The samples were separated by SDS-PAGE and analyzed by western blot.

### Immunofluorescence staining and colocalization analysis

LLC-PK1 cells were grown in glass coverslips for 24 h, then, co-transfected with plasmids encoding the chaperone RAP and mMeg using Lipofectamin 2000. For the colocalization experiments wild-type mMeg, mMeg S/D or mMeg S/A were used. To visualize Rab11 the receptor was co-transfected with mCherry -Rab11. After 24 h of expression, cells were treated with 50 μM YU142670 or vehicle for 4 h. Cells were fixed with 4% paraformaldehyde in PBS and then permeabilized with 0.2% Triton X-100 in PBS. Next, the cells were blocked with 5% BSA in PBS and incubated successively with the primary antibodies (anti-HA and anti-EEA1 or anti-Rab7) and the corresponding secondary antibodies. Images were captured by Inverted Nikon Ti2-E and deconvolved with DeconvolutionLab (Sage et al., 2017) Manders coefficient was calculated with JaCoP (Bolte and Cordelières, 2006), a plugin for ImageJ (NIH). Briefly, images of cells with the two stains were selected and separated. Cells were analyzed with the JaCoP function of Manders’ Coefficient and data was stored for analysis.

### Determination of megalin ectodomain secretion

Silenced or control cells were seeded at a density of 8 × 10^5^ and grown in DMEM plus 10% SBF for 48 h to accumulate the proteolytic fragment of mMeg in the culture media. For matrix metalloproteinases inhibition (MMPi) treatment, cells were treated with or without 10 μM GM6001 for 48 h, replenishing every 12 h. The medium was collected, and the cells were lysed. The media were clarified by centrifugation at 10,000 *g* for 10 min and then concentrated with Centricon^®^ 100. Proteins from the concentrated medium were immunoprecipitated with anti-HA antibody coupled with protein G–agarose beads (Pierce). Samples were separated by SDS-PAGE and analyzed by Western blot.

### Flow cytometry

For measurements of total mMeg, cells expressing the mini receptor were grown on 100-mm dishes until 80% confluency. Cells were detached with PBS containing 5 mM EDTA and permeabilized with 0.75% saponin in PBS before incubation with Alexa488-conjugated anti-HA antibody. The results were displayed as mean fluorescence per cell. For measurements of surface mMeg, non-permeabilized cells were detached with PBS containing 5 mM EDTA and incubated with monoclonal Alexa488-conjugated anti-HA antibody. Cells from another set were permeabilized with 0.75% saponin in PBS and incubated with anti-HA-488 to measure the total amount of receptors. Results were plotted as surface (non-permeabilized cells) mean fluorescence *versus* total (permeabilized cells) mean fluorescence. Background fluorescence intensity was assessed without the primary antibody and subtracted from mean fluorescence. Mean fluorescence values were obtained in triplicate with a FACScalibur (BD Biosciences-Pharmingen, Sweden), and the data were analyzed with the Weasel software.

### Phosphorylation assay and insulin treatment

The strategy to measure phosphorylation was described before (Li et al., 2000; Yuseff et al., 2007). Briefly, seeded cells were washed and incubated twice with phosphate-free minimal essential medium (Gibco) for 20 min, followed by the addition of 200 uCi/ml of [^32^P]-orthophosphate at 37°C for 2 h. Then, cells were washed and lysed in a PBS buffer containing 1% Triton X-100, 1 mM glycerophosphate, 1 mM sodium orthovanadate, 5 mM sodium fluoride, and protease inhibitors. For insulin treatment, the protein was included at a final concentration of 100 nM in all steps of the phosphorylation assay. Following immunoprecipitation with anti-megalin antibody, samples were examined *via* SDS–PAGE and autoradiography. The percentage of megalin phosphorylation was calculated by densitometry using ImageJ and normalized to the levels of mMeg detected by western blot from an aliquot of cell lysate.

### Cell-surface biotinylation

To determine cell surface mMeg localization, the cells were biotinylated as described (Marzolo et al., 2003). Briefly, control or OCRL1 silenced cells were serum-starved for 2 h and incubated with 100 nM insulin for 2 or 4 h. The cells were washed in ice-cold PBS and biotinylated at 4°C with 0.5 mg/ml sulfo-NHS-LC- biotin for 1 h. Then, the cells were washed with PBS and the biotin was quenched with 50 mM NH_4_Cl for 10 min. Cells were lysed and biotinylated cell-surface proteins were adsorbed to streptavidin agarose beads for 2 h at 4°C in rotation. Beads were washed, boiled in Laemmli sample buffer and the proteins of interest were separated by SDS-PAGE and analyzed by western blot.

### Insulin signaling

Cells were serum-starved for 4 h with or without 50 μM YU142670 (Pirruccello et al., 2014). Next, cells were incubated with 100 nM insulin (with or without 50 μM YU142670), to promote AKT and GSK3β phosphorylation due to insulin signaling. Cells were washed twice with PBS, lysed, and analyzed by western blot.

### Statistical analysis

The blots were quantified with the ImageJ software, and qPCR analyses were performed using a relative quantification mathematical model, as previously described (Pfaffl, 2001). Data were expressed as the mean ± SEM (standard error of the mean) from at least three independent experiments. Comparisons of two conditions were performed using Student’s t-test or Mann-Whitney. For multiple comparisons data was analyzed using ANOVA with Bonferroni and Dunnett´s correction. The statistical analyses and graphs were performed using GraphPad Prism 5.

## Results

### Megalin is post-transcriptionally regulated in a cellular model of Lowe Syndrome

**FIGURE 1:**
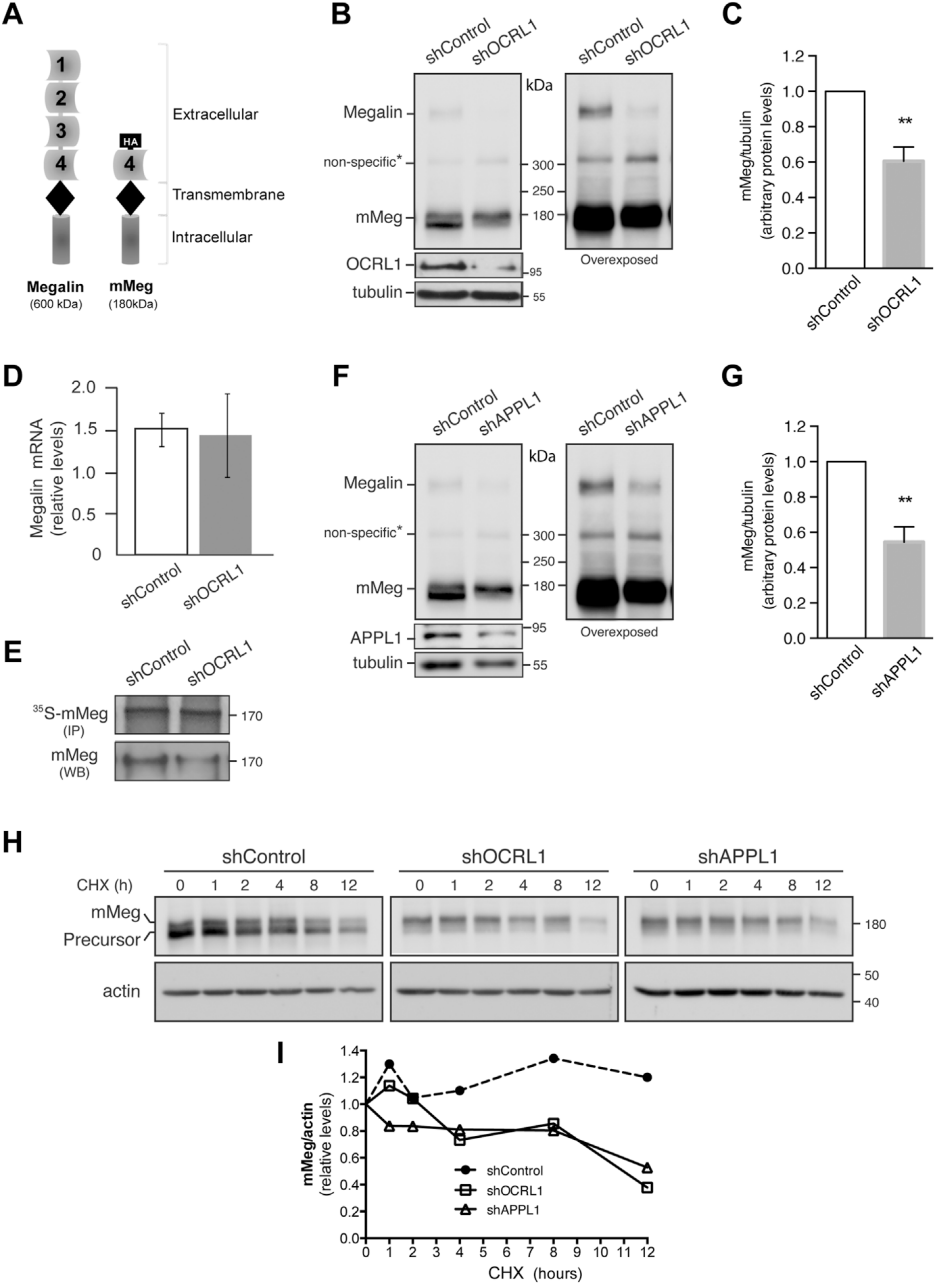
Megalin is down-regulated in OCRL1 and APPL1 KD proximal tubule cells. **(A)** Schematic representation of the full-length megalin with its four ligand-binding domains and the mini receptor with the fourth ligand-binding domain tagged with HA-epitope (mMeg). **(B–H)** LLC-PK1 cells stably expressing mMeg (mMeg-LLC-PK1) were infected with non-target shRNA (shControl), shRNA directed against OCRL1 (shOCRL1) or APPL1 (shAPPL1) lentiviruses. **(B)** Endogenous megalin and mMeg were analyzed in whole-cell lysates of control and OCRL1 silenced cells by western blot. **(C)** Quantification of mMeg protein levels corrected with tubulin levels in control and OCRL1 KD cells. Data are expressed as the means ± SEM of N = 5 independent experiments (*t*-test, **p < 0.01). **(D)** Quantitative PCR was used to analyze mRNA levels of megalin. Three independent assays were performed, and the average ±SD was plotted on the graph. **(E)** Metabolic labeling of control or OCRL1 silenced cells with [^35^S]-methionine/cysteine for 4 h mMeg was immunoprecipitated (IP) and analyzed by autoradiography. Additionally, an aliquot of the whole-cell lysate was used to total mMeg by western blot (WB). **(F)** Endogenous megalin and mMeg were analyzed in whole-cell lysates of control and APPL1 silenced cells by western blot. **(G)** Quantification of mMeg protein levels corrected with tubulin levels in control and APPL1 KD cells. Data are expressed as the means ± SEM of five independent experiments (*t*-test, **p < 0.01). **(H)** Control or silenced cells were treated with CHX (100 μM), and the expression of mMeg, Precursor-mMeg, and actin were determined by western blot at indicated times. **(I)** Graph corresponds to mMeg protein levels corrected with actin levels in control and silenced cells.

It has been proposed that the defect in re-absorption function and secretion of megalin in LS result from receptor trafficking deregulation (Norden et al., 2002; Vicinanza et al., 2011). As was reported, the lack of OCRL1 does not affect megalin apical distribution or its internalization, but the recycling of the receptor is significantly reduced in both proximal and distal epithelial tubular cells (Vicinanza et al., 2011). To study megalin expression and trafficking, we silenced OCRL1 in the porcine kidney proximal tubule cells LLC-PK1. In this model, we evaluated the protein levels of the receptor, both endogenous megalin and the megalin mini receptor, mMeg, an accepted model to study trafficking and phosphorylation of the full-length receptor (Yuseff et al., 2007; Farfán et al., 2013) (Figure 1A). Interestingly we observed a significant decrease in megalin expression, at the protein level, in OCRL1 silenced cells (Figures 1B,C). On the other hand, and in line with findings in zebrafish (Oltrabella et al., 2015), the reduction of megalin protein levels in OCRL KD cells was not a result of a transcriptional regulation because megalin mRNA expression, evaluated by quantitative PCR, did not change (Figure 1D). Besides, the amount of mMeg found after a pulse of ^35^S-methionine was similar in control and OCRL1 KD cells (Figure 1E), confirming that the decreased total levels of the receptor were not due to a reduction in its biosynthesis. These results suggest that OCRL1 regulates megalin at a post-transcriptional level in the proximal kidney cell line.

OCRL1 and the endosomal protein APPL1 are present in endocytic vesicles (Erdmann et al., 2007). The recruitment of OCRL1 to the phagosomes and endosomes is regulated by Rab proteins, including Rab5, Rab22a, and Rab35 (Hyvola et al., 2006; Fukuda et al., 2008; Noakes et al., 2011). Besides, the association of OCRL1 to endosomes is indirectly regulated by APPL1 by its interaction with Rab5 (McCrea et al., 2008; Bohdanowicz et al., 2012) and APPL1 interacts with megalin (Erdmann et al., 2007). Previous results showed that in OCRL KD cells, the presence of megalin in APPL1-positive endosomes is reduced, whereas its presence in EEA1-positive endosomes is increased (Vicinanza et al., 2011; Festa et al., 2019). However, the role of APPL1 in megalin expression has not been evaluated. Therefore, we determined whether silencing APPL1 modifies megalin levels. Our findings indicate that this was the case; as is shown in Figures 1F,G, megalin protein levels were significantly reduced in APPL1 KD cells. To explore if the reduction of OCRL1 and APPL1 decreases megalin by promoting its degradation, the receptor half-life was evaluated by measuring its disappearance over time in control and OCRL1 or APPL1 KD cells. Cells were treated with cycloheximide (CHX) for up to 12 h. The disappearance of megalin in OCRL1 and APPL1 silenced cells was faster than in control cells (Figures 1H,I). These results underscored the roles of OCRL1 and APPL1 in megalin protein stability.

In control cells treated with CHX, the inhibition of proteasome by epoxomycin partially recovered megalin levels but the inhibition of lysosome, with NH4Cl, had no effect (Supplementary Figure S1A,B). In OCRL1 silenced cells the recovery of megalin levels was significant upon proteasome inhibition. Compared with the control cells, the inhibition of lysosome in shOCRL1 cells had a slight but not significant effect. These results suggest that the lack of OCRL1 induces megalin degradation by the proteasome and possibly in less extension by the lysosome. APPL1 silenced cells also show a more predominant role of proteasome than lysosome in megalin degradation.

### Megalin proteolytic processing, ectodomain secretion and surface levels are decreased in OCRL1 and APPL1 knock-down cells

LS patients have reduced megalin levels in the urine (Norden et al., 2002; Suruda et al., 2017). Besides the reduction in total megalin levels, explained by increased receptor degradation, a possible mechanism involved in the decrease of megalin in the urine of LS patients is related to alterations in the proteolysis of the receptor ectodomain. Endogenous megalin is first proteolyzed by matrix metalloproteinases (MMPs), stimulated by PKC; this process is followed by a *γ*-secretase mediated intramembrane processing of the resulting megalin C-terminal fragment; this sequential proteolytic processing has been documented in opossum renal cell line (OK) (Zou et al., 2004) as well in LLC-PK1 cells (Cabezas et al., 2019) among others. To evaluate the proteolytic processing in our system, we first characterized and compared the processing of mMeg, expressed in LLC-PK1 and MDCK renal epithelial cells. We determined the distribution of the extracellular and intracellular fragments of mMeg since a differential localization of these domains originated from endogenous megalin in rat proximal tubule epithelium was previously reported (Bachinsky et al., 1993; Zou et al., 2004). In MDCK cells mMeg extracellular and cytoplasmic domains were not always colocalizing within the cell (Supplementary Figure S2A,B), consistent with constitutive processing of the receptor as occurs with other members of the family (Larios and Marzolo, 2012). Moreover, it was also possible to observe that, in addition to the detection of N- and C-terminal megalin in the same structure, amino and carboxy-terminal fragments were also found separated (Supplementary Figure S2B), corroborating the observations in renal tissues analyzed by electron microscopy (Bachinsky et al., 1993; Zou et al., 2004).

**FIGURE 2:**
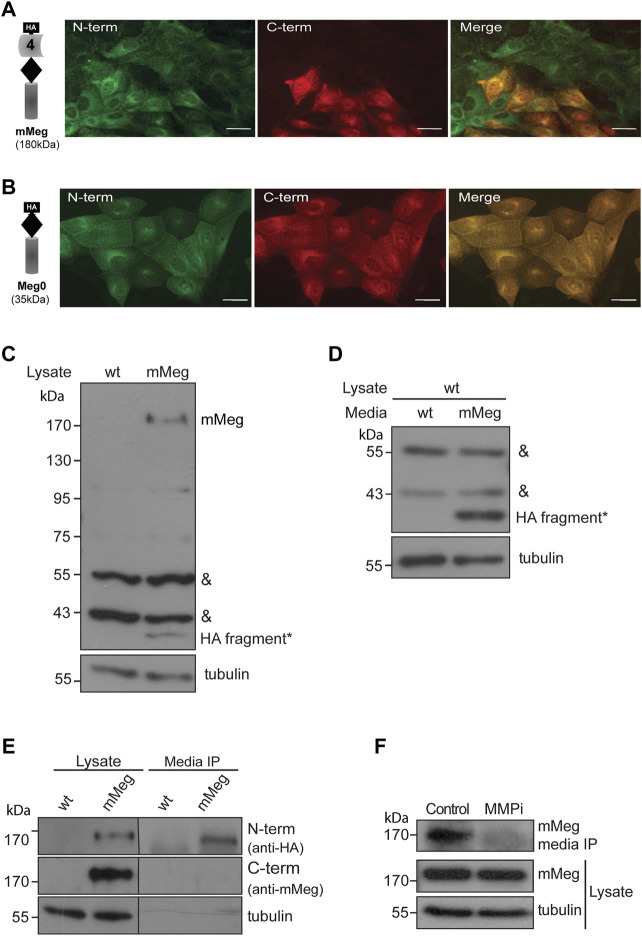
Characterization of megalin intracellular and secreted fragments. Immunofluorescence of LLC-PK1 cells stably expressing **(A)** mMeg or **(B)** Meg0 to detect amino-terminal (N-term) or carboxy-terminal (C-term) domains of the receptor with anti-HA (green) and anti-Megalin (red) antibodies, respectively. The images were acquired by epifluorescence microscopy. Scale bar, 10 µm. **(C)** Whole-cell lysates of wt LLC-PK1 and mMeg-LLC-PK1 cells were analyzed by western blot using anti-HA to detect mMeg or its N-term fragments. Symbols correspond to a non-specific band (&) and the low molecular weight band exclusive to the mMeg expressing cell lysate (*). **(D)** Whole-cell lysates of wt LLC-PK1 cells were incubated with conditioned media of either wt or mMeg -LLC-PK1 cells, analyzed by western blot using an anti-HA antibody, which recognizes the N-terminal portion of mMeg. Symbols correspond to the non-specific band (&) and low molecular weight band found in the cell lysate of wt LLC-PK1 cells incubated with conditioned media from mMeg-expressing LLC-PK1 cells (*). **(E)** mMeg-LLC-PK1 cells were grown to confluence for 48 h. The conditioned medium was concentrated and then immunoprecipitated with an anti-HA antibody. Protein samples, immunoprecipitated media, and cell lysates were analyzed with an anti-HA antibody to detect megalin ectodomain (N-term) and with an anti-megalin cytoplasmic domain polyclonal antibody to detect megalin carboxy-terminal fragment (C-term). Tubulin was used as a loading control. **(F)** mMeg expressing cells were incubated with vehicle (Control) or 10 μM MMPi (GM6001, general matrix metalloprotease inhibitor) for 48 h and replenished every 12 h. The immunoprecipitated media and cell lysates were analyzed by western blot to detect mMeg and tubulin as load control.

Furthermore, by immunofluorescence, we found an interesting distribution pattern of the receptor in LLC-PK1 cells overexpressing mMeg (in which minimegalin levels are several times higher than endogenous megalin, see Figure 1B); some cells exhibited the label corresponding to both domains while in others, only the label corresponding to the ectodomain (N-terminal, containing HA epitope) was detected (Figure 2A). Besides, this dual pattern was not observed in cells expressing mMeg0, a mini receptor lacking the extracellular domain but including an amino-terminal HA-epitope (Figure 2B). This result suggests that the cells exclusively positive for the ectodomain staining (Figure 2A) could capture the N-terminal part of the receptor from the media. Accordingly, western blot evaluation of the cellular proteins showed a band recognized by the anti-ectodomain antibody (anti-HA), of around 40 kDa, only present in lysates from mMeg transfected cells (Figure 2C). To test if this band corresponds to an endocytosed minimegalin N-terminal fragment, we incubated non-transfected cells with the conditioned media obtained either from wild-type or mMeg expressing cells. The result shows a band, 40 kDa, only detected in lysates of the cells exposed to the conditioned media of mMeg expressing cells (Figure 2D). Overall, these observations suggest that megalin ectodomain secretion would be followed by the internalization of this domain, or a fragment of it, in our cellular model.

Then, we evaluated the conditioned media of LLC-PK1 mMeg cells. We detected a fragment of mMeg recognized only by anti-HA but not by an anti-C terminal antibody, indicating that this soluble N-terminal fragment of the protein probably results from a shedding process. This band is slightly smaller than the one present in cell lysates, corresponding to the full-length mMeg (Figure 2E). Similar results were found in mMeg expressing MDCK cells (Supplementary Figure S3), indicating that megalin is secreted to the culture media as a proteolytic product. Similar to the endogenous receptor, matrix metalloproteinases (MMPs) and a disintegrin and metalloproteinase domain-containing (ADAM) proteins would be involved in the shedding of mMeg because the inhibition of these enzymes with the general inhibitor GM6001 reduced the secretion of the receptor ectodomain (Figure 2F).

**FIGURE 3:**
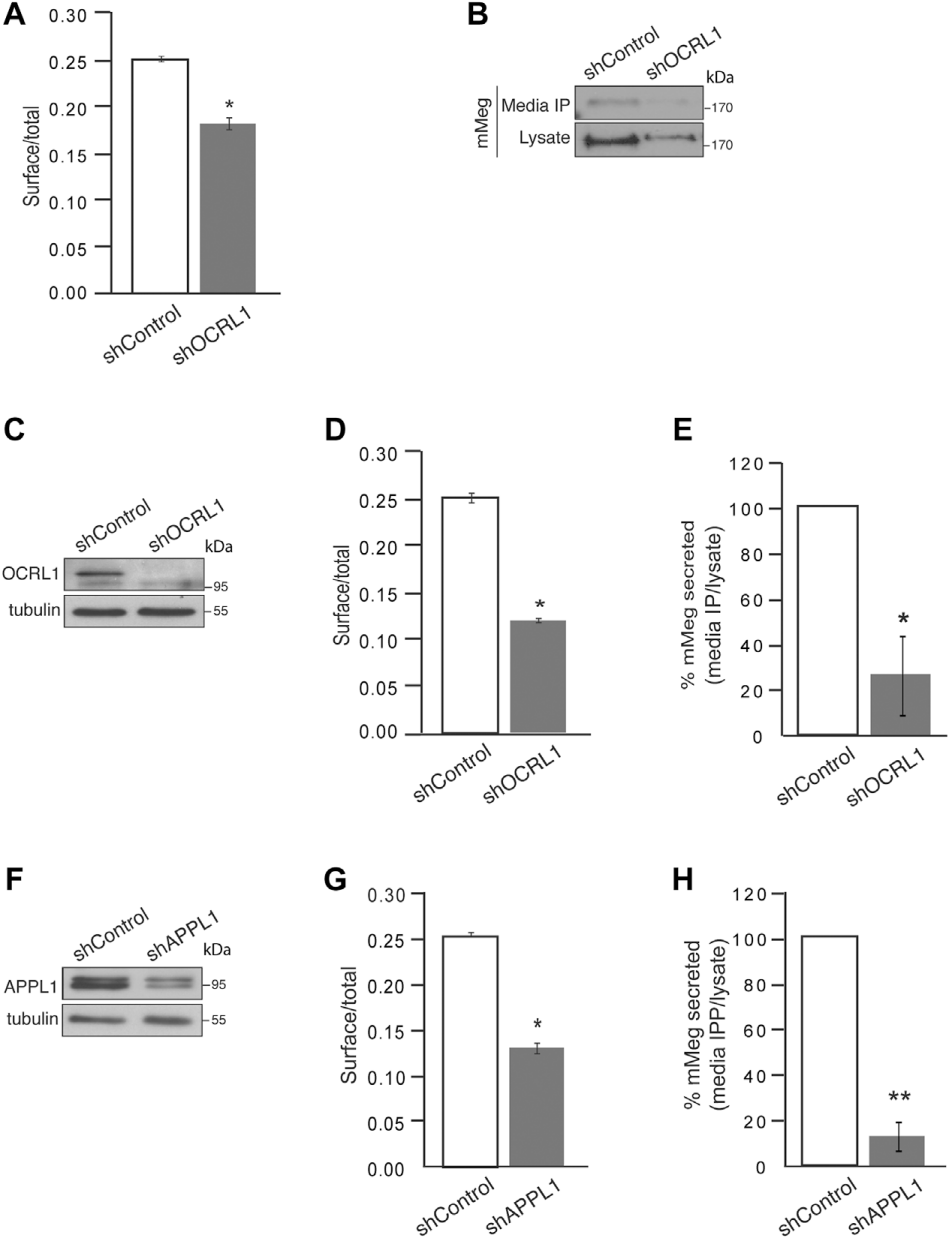
Cell surface megalin distribution and extracellular domain release decrease under OCRL1 and APPL1 silencing. **(A)** mMeg-LLC-PK1 cells, non-permeabilized or permeabilized, control, or OCRL1-silenced were incubated with an anti-HA-488-conjugated antibody and analyzed by flow cytometry. The results are plotted as the ratio of expression levels observed in non-permeabilized (surface) *versus* permeabilized cells (total). Data are expressed as the means ± SEM of N = 3 independent experiments (*t*-test, *p < 0.05). **(B)** Conditioned media of control or OCRL1 silenced mMeg-LLC-PK1 cells were processed by anti-HA immunoprecipitation (Media IP). Samples of IP and cell lysates were analyzed by western blot with an anti-HA antibody to detect the megalin ectodomain (N-term). **(C–H)** MDCK cells stably expressing mMeg (mMeg-MDCK) were infected with non-target shRNA (shControl), shRNA directed against OCRL1 (shOCRL1) or APPL1 (shAPPL1) lentiviruses. **(C)** Protein levels of OCRL1 were analyzed in whole-cell lysates of control and OCRL1 silenced cells by western blot. **(D)** Flow cytometry analyzes mMeg surface localization in control and OCRL1 silenced cells. The plot shows the surface vs. total ratio of expression levels. Data are expressed as the means ± SEM of N = 3 independent experiments (*t*-test, *p < 0.05). **(E)** Analysis of immunoprecipitated conditioned medium and cell lysates of control or OCRL1 silenced cells by western blot with an anti-HA antibody. The plot shows the levels of mMeg in the immunoprecipitated media corrected by total. Data are expressed as the means ± SEM N = 3 independent experiments (*t*-test, *p < 0.05). **(F)** Protein levels of APPL1 were analyzed in whole-cell lysates of control and APPL1 silenced cells by western blot. **(G)** Flow cytometry analyzes mMeg surface localization in control and APPL1 silenced cells. The plot shows the surface vs. total ratio of expression levels. Data are expressed as the means ± SEM of N = 3 independent experiments (*t*-test, *p < 0.05). **(H)** Analysis of immunoprecipitated conditioned medium and cell lysates of control or APPL1 silenced cells by western blot with an anti-HA antibody. The plot shows the levels of mMeg in media IP corrected by total. Data are expressed as the means ± SEM N = 3 independent experiments (*t*-test, **p < 0.01).

To determine if megalin processing is altered in LS cellular models, we evaluated the cell surface expression and secretion of mMeg, to the culture media, in OCRL1 and APPL1 KD cells. As previously found in HK2 cells (Vicinanza et al., 2011), the reduction of OCRL1 in LLC-PK1 cells induced a significant decrease in mMeg levels at the cell surface (Figure 3A). The ectodomain of megalin in the media was also evidently reduced in this proximal tubule cell (Figure 3B). Similar results were observed in OCRL1 KD MDCK cells (Figures 3C–E). These results highlight a role for OCRL1 in regulating megalin surface expression, proteolysis, and ectodomain secretion.

Additionally, we evaluated whether the previous relationship between OCRL and APPL1 exists in our model, specifically if the effects on mMeg trafficking observed in OCRL1 silenced cells replicate under APPL1 silencing conditions (Figure 3F). Our results show a significant reduction in megalin cell surface expression in APPL1 silenced cells (Figure 3G). Interestingly, APPL1 silencing also significantly reduced mMeg secretion to the culture media (Figure 3H). The similarity of the effects of APPL1 KD with OCRL1 KD again suggests a link between these two endosomal proteins in regulating mMeg trafficking and processing.

### OCRL1 regulates the phosphorylation and the endosomal distribution of megalin

**FIGURE 4:**
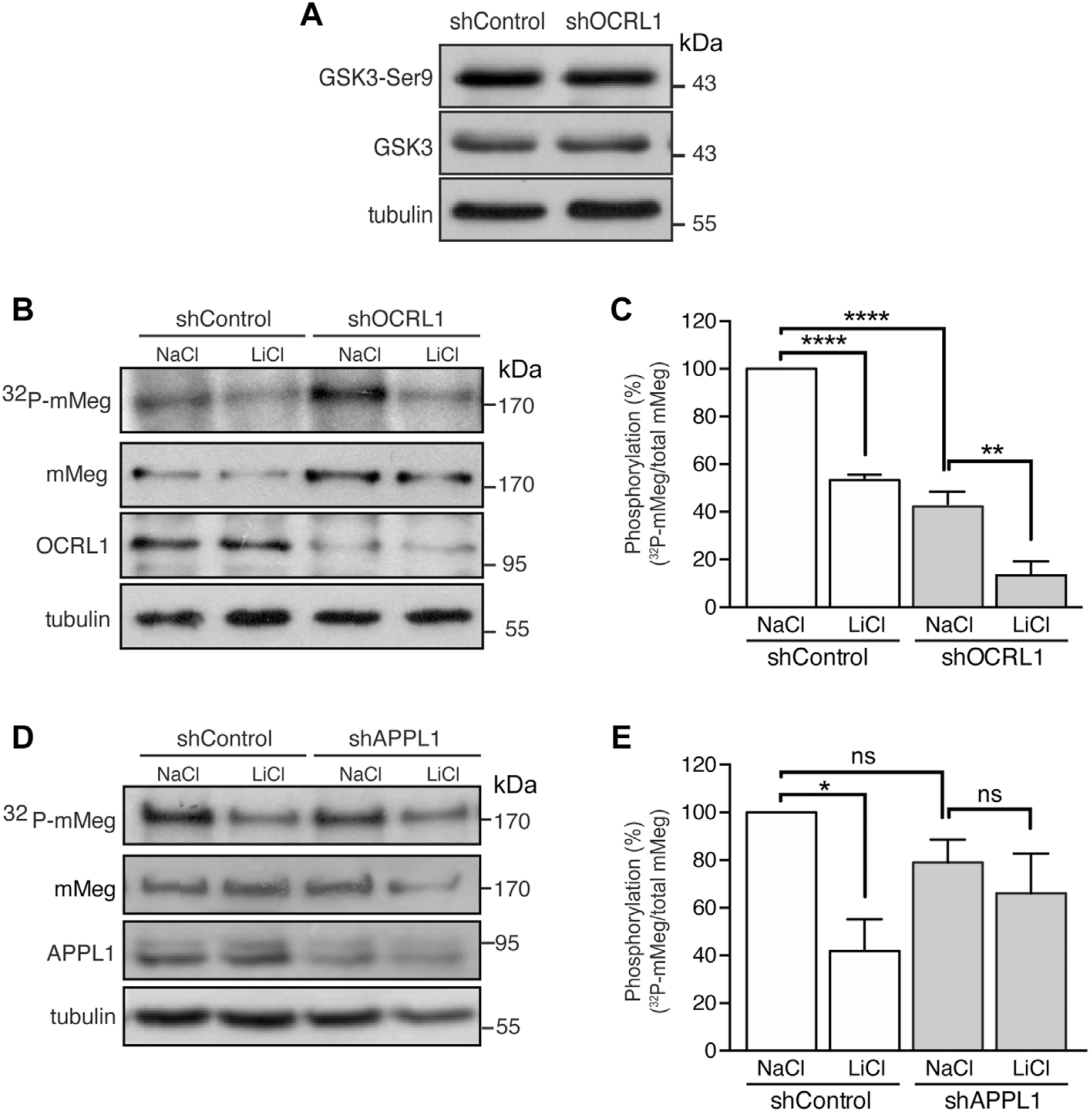
Reduced mMeg phosphorylation in mMeg-MDCK OCRL1 silenced cells. **(A)** Control or OCRL1 silenced cells were lysed and analyzed by western blot with antibodies against phosphorylated GSK3ß (Ser9) and total GSK3ß. **(B)** Control or silenced for OCRL were treated with LiCl (50 mM) or NaCl (50 mM) as control. For phosphorylation assays, the cells were incubated with [^32^P]-orthophosphate for 2 h at 37°C, followed by immunoprecipitation of megalin from cell lysates using an anti-megalin cytoplasmic domain. The immune complexes were analyzed by SDS-PAGE and visualized by autoradiography. Aliquots of whole-cell lysates were used to detect mMeg by western blot. **(C)** Graph shows the percentage of phosphorylated mMeg related to the total. Data are expressed as the means ± SEM of N = 3 independent experiments (ANOVA, ****p < 0.0001, **p < 0.01). **(D)** Control or silenced for APPL1 were treated with LiCl (50 mM) or NaCl (50 mM) as control. For phosphorylation assays, the cells were incubated with [^32^P]-orthophosphate for 2 h at 37°C, followed by immunoprecipitation of megalin from cell lysates using an anti-megalin cytoplasmic domain. The immune complexes were analyzed by SDS-PAGE and visualized by autoradiography. Aliquots of whole-cell lysates were used to detect mMeg by western blot **(E)** Graph corresponds to the percentage of phosphorylated mMeg related to the total of control or APPL1 silenced cells. Data are expressed as the means ± SEM of N = 3 independent experiments (ANOVA, *p < 0.05).

Megalin trafficking is particularly affected at the early endosomes in OCRL1 silenced cells due to inefficient recycling back to the plasma membrane, partly due to an accumulation of actin filaments around the endosomes (Vicinanza et al., 2011). On the other hand, we have described that megalin’s recycling and cell surface levels are inhibited by GSK3-mediated phosphorylation of its cytoplasmic domain (Yuseff et al., 2007). Thus, we wondered whether GSK3β-mediated megalin phosphorylation could be increased in OCRL1 KD cells as an additional explanation for its reduction at the cell surface. To address this possibility, we evaluated megalin phosphorylation in mMeg-MDCK silenced for OCRL1 (Vicinanza et al., 2011). In OCRL1 KD cells, protein levels and the basal activation of GSK3ß, analyzed by phosphorylation of its inhibitory residue Ser9, were not changed (Figure 4A). Then, we evaluated the phosphorylation of mMeg by metabolic labeling with ^32^P-orthophosphate, as described (Yuseff et al., 2007). Contrarily to our initial idea, in OCRL1 KD cells megalin phosphorylation was reduced to 59% of the control cells (Figures 4B,C), something unexpected considering that the basal inhibition GSK3ß (phosphorylated in Ser 9) was similar in both wild-type and OCRL1 KD cells. Part of the remaining phosphorylated megalin was GSK3-dependent as LiCl, a GSK3 inhibitor (Serretti et al., 2009), diminished even more the phosphorylation of the receptor (Figures 4B,C). Overall, these results indicate that in our LS cellular model, the reduced surface expression of megalin is not explained by an increase in its GSK3ß-mediated phosphorylation. In contrast with the effect due to the lack of OCRL1, silencing APPL1 did not significantly affect the basal levels of megalin phosphorylation, although there was a reduction trend (Figures 4D,E).

One possibility to explain a lower megalin phosphorylation in LS cells is that GSK3ß has less access to its substrate. To test this possibility, we determined the colocalization of endogenous megalin and transfected GSK3-HA in LLC-PK1 cells. Cells were treated with YU142670, an inhibitor of OCRL1 (Pirruccello et al., 2014), or vehicle, and the colocalization of megalin with GSK3ß was analyzed after the immunodetection of both proteins. The blocking effect of the inhibitor was assessed by determining the size of the EEA1 positive endosomes (Supplementary Figure S4A,B). The results show megalin and GSK3ß colocalize similarly in control and inhibitor-treated cells (Supplementary Figure S4C,D), a result that does not support the option of decreased substrate-kinase encounter in LS conditions.

**FIGURE 5:**
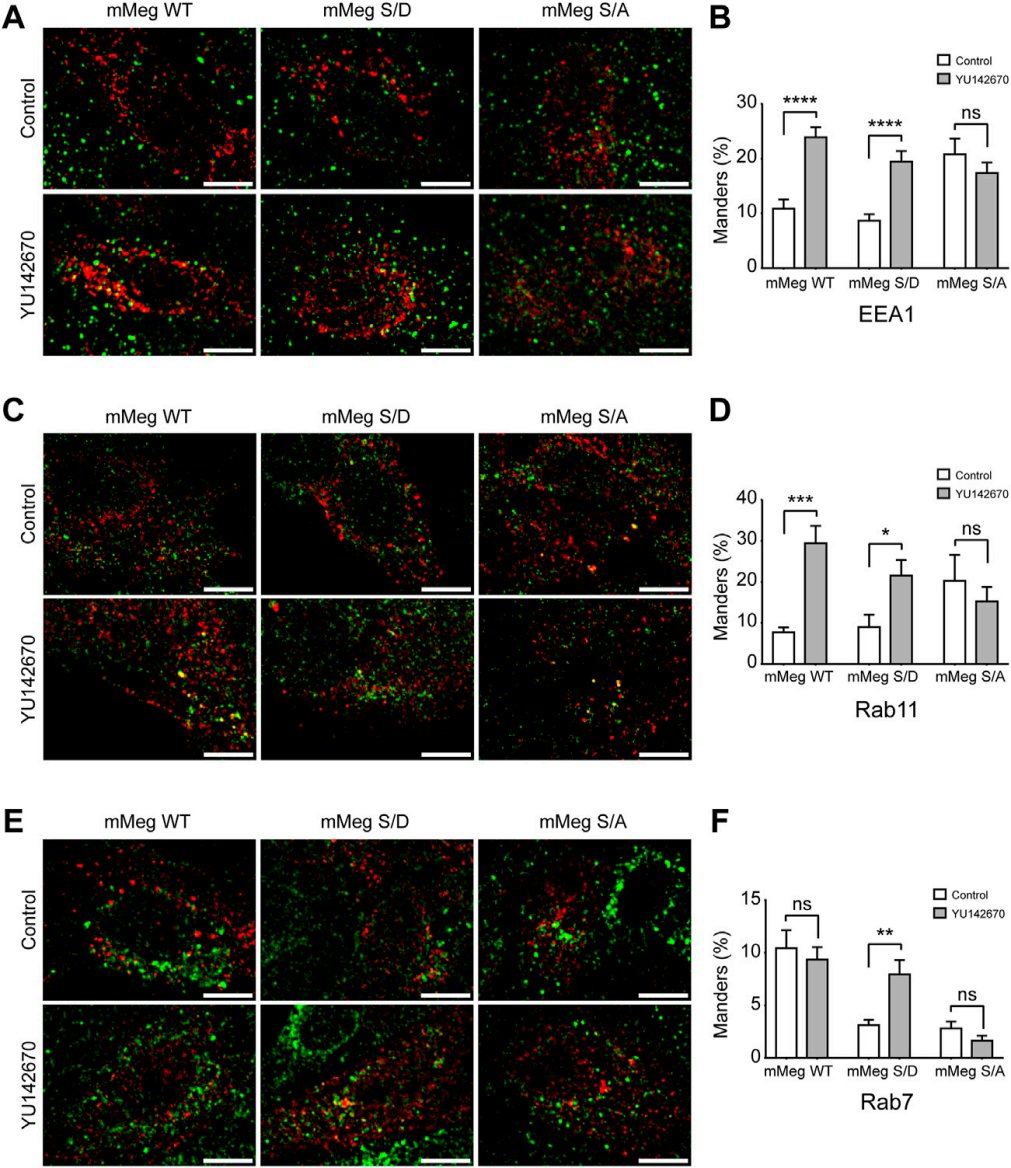
Endosomal distribution of megalin and its phosphorylated and non-phosphorylated forms in control and OCRL inhibition conditions. LLC-PK1 were transfected with HA-tagged minimegalins (mMeg) wt, phosphomimetic (mMeg S/D) or non-phosphorylatable mMeg S/A. Then, cells were treated with 50 μM YU142670 or vehicle for 4 h, fixed and processed for fluorescence microscopy. Preparations were imaged with Confocal Nikon Timelapse. Scale Bars 10 μM. **(A)** Cells were stained with anti-EEA1 (green) and anti-HA (red). **(B)** At least 34 separated cells were analyzed with ImageJ to measure the Manders percentage between EEA1 endosomes and HA-tagged mMeg structures. ANOVA, ****p < 0.0001. **(C)** LLC-PK1 cells were co-transfected with mCherry-Rab11 and HA-tagged mMeg. Then, cells were treated with 50 μM YU142670 for 4 h, fixed and processed to detect mMeg (HA, red) and Rab11 (green). The color was changed for consistency with the other panels. **(D)** At least 12 separated cells were analyzed with ImageJ to measure the Manders percentage between Rab11-positive endosomes and mMeg structures. ANOVA, ***p < 0.001, *p < 0.05. **(E)** LLC-PK1 transfected with HA-tagged mMeg. Then, cells were treated with 50 μM YU142670 for 4 h, fixed and processed to detect mMeg (HA, red) and Rab7 (green). **(F)** At least 18 separated cells were analyzed with ImageJ to measure the Manders percentage between Rab7-positive endosomes and mMeg structures. ANOVA, **p < 0.01.

Then, we asked if megalin is differentially distributed in the endosomal compartments dependent on phosphorylation status and how this localization could be changed if OCRL1 activity is decreased. To have insights into the endosomal distribution of megalin in its phosphorylated and non-phosphorylated forms, the wild-type having the cytoplasmic PPPSP motif recognized by GSK3ß, the phosphomimetic PPPDP (S/D) and the non-phosphorylated PPPAP (S/A) forms of mMegs (Yuseff et al., 2007; Marzolo and Farfán, 2011) were expressed in LLC-PK1 treated or not with YU142670. The steady-state distribution of mMeg was determined by colocalization with EEA1, Rab11 and Rab7 under control conditions (Figures 5A–F). Due to the lack of specific staining of the Rab11 antibody in LLC-PK1 cells, the distribution of the megalin in the recycling compartment was assessed upon transfection of mCherry-Rab11. In YU142670 treated cells, the wild-type and S/D mutant mMegs were significantly increased in EEA1 positive early endosomes (Figures 5A,B) and in Rab11 recycling endosomes (Figures 5 C, D). In contrast, upon the inhibition of OCRL1 only the mMeg S/D mutant was increased in Rab7 positive endosomes, even though the wild-type mMeg was more present in this compartment under control conditions (10.8% wt vs. 3.1% S/D) (Figures 5 E, F). Interestingly, the mMegS/A that is not a substrate of GSK3ß was rather refractory to the reduction of OCRL1 activity. This mutant has a fast-cycling behavior (Yuseff et al., 2007), something evident when its presence in the different endosomal compartments is analyzed under control conditions; compared with the wild-type mMeg, the mMeg S/A mutant was more present in early/recycling endosomes (18.5% wt; 41.05% S/A) and less in late Rab7 positive endosomes (10.4 wt% vs. 3.1% S/A). Overall, these pieces of evidence suggest that the endosomal trafficking of megalin is differentially affected by the reduction in the activity of OCRL1 and the phosphorylation status of the receptor.

It is important to consider that the increased presence of megalin in the Rab11-endosomal compartments in YU142670 treated cells probably reflects that the inhibition of megalin recycling also takes place from a more mature recycling compartment and not only from an EEA1-sorting endosome. Moreover, our data also show that the inhibition of OCRL activity affects the identity of the endosomal compartments; cells treated with YU142670 showed a significant increase in the colocalization of EEA1 with Rab11 (Supplementary Figure S4E,F).

### Insulin treatment decreases megalin phosphorylation and increases megalin at the cell surface despite a reduction in the signaling pathway in OCRL1-and APPL1-silenced cells

**FIGURE 6:**
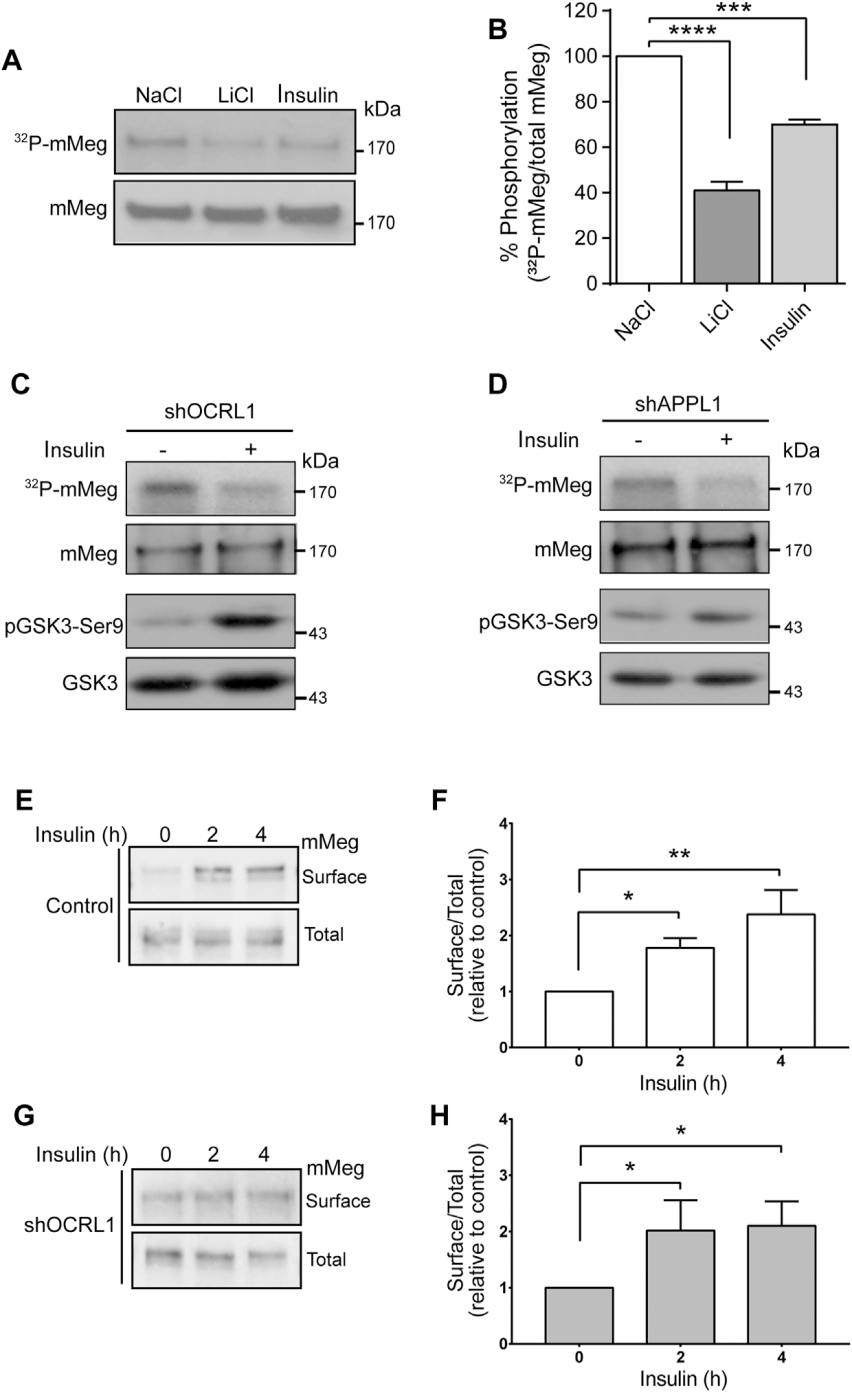
Insulin decreases megalin phosphorylation and increases megalin surface expression in control and LS cellular models. **(A)** mMeg-LLC-PK1 cells treated with NaCl (50 mM), LiCl (50 mM) or insulin (100 nM) were labeled [^32^P]-orthophosphate for 2 h at 37°C, followed by immunoprecipitation of megalin with an anti-megalin cytoplasmic domain. The immune complexes were analyzed by SDS-PAGE and visualized by autoradiography. Aliquots of whole-cell lysates were used to detect mMeg by western blot. **(B)** Graph corresponds to the percentage of phosphorylated mMeg related to the total of NaCl, LiCl or insulin-treated cells. Data are expressed as the means ± SEM of N = 3 independent experiments (ANOVA, ****p < 0.0001, ***p < 0.001). Phosphorylation assays were performed in OCRL1 **(C)** or APPL1 **(D)** silenced cells in the presence or the absence of 100 nM insulin. Immunoprecipitated radiolabeled mMeg was evaluated by autoradiography. Total mMeg and GSK3ß (total and phosphorylated forms) were detected by western blot. **(E,F)** Control or **(G,H)** OCRL1 silenced cells were serum-starved for 2 h before the incubation with 100 nM insulin by the indicated period of time. Cells were biotinylated to determine the surface levels of mMeg. The whole lysates were used for total receptor levels. Samples were analyzed by western blot. The graphs shows surface vs. total ratio of mMeg expression levels relative to control time 0. N = 4; ANOVA, *p < 0.05, **p < 0.01.

Insulin increases megalin levels in cell culture under conditions resembling hypertension and type-2 diabetes, both associated with chronic kidney disease (Hosojima et al., 2009; Bryniarski et al., 2018). Besides, insulin inhibits GSK3 (Cross et al., 1995). As mentioned before, megalin’s recycling from endosomes to the cell surface is reduced by GSK3-mediated phosphorylation of its cytoplasmic domain, decreasing the amount of megalin available for ligand binding (Yuseff et al., 2007). Therefore, insulin could mediate a physiological way to inhibit megalin phosphorylation and, eventually, increase the receptor surface levels. First, we tested the effect of 100 nM insulin on megalin phosphorylation in LLC-PK1 cells (Figure 6). Interestingly, 4 h of insulin treatment significantly reduced megalin phosphorylation up to 60% of the control (Figures 6A,B). Moreover, in OCRL1 and APPL1 KD cells, insulin was still able to reduce megalin phosphorylation as well as GSK3ß activity, measured by the PKB/AKT-mediated phosphorylation in Ser9 (Figures 6C,D).

Accordingly, insulin treatment of both wild-type and LS cells (OCRL KD) significantly increased megalin surface expression after 4 h of treatment (Figures 6E–H). These results suggest that insulin, *via* inhibition of GSK3ß -mediated megalin phosphorylation, could promote more efficient recycling of the receptor in normal and in OCRL1 dysfunction conditions.

**FIGURE 7:**
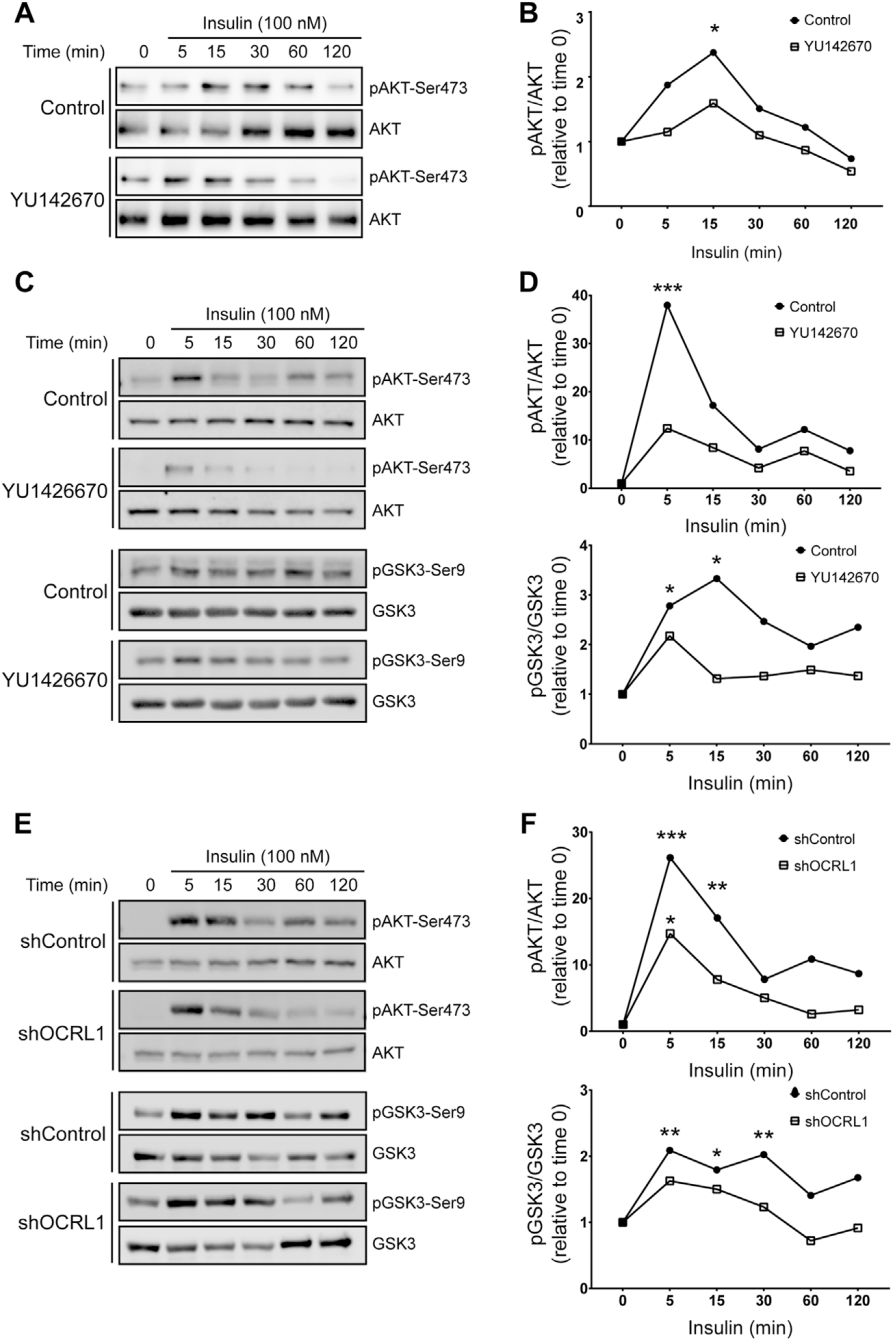
Insulin signaling is decreased in LS cellular models. mMeg-LLC-PK1 **(A–B)** or HeLa **(C–D)** cells were serum starved for 4 h ± 50 μM YU1426670 and then incubated with 100 nM insulin ± 50 μM YU1426670 for indicated periods of time. **(A,C)** Cell lysates were analyzed by western blot to detect total and phosphorylated forms of AKT. **(B)**. Graph corresponds to the protein levels of phosphorylated AKT corrected by total levels of AKT relative to time 0. N = 3; ANOVA, *p < 0.05.**(D)** Graph corresponds to the protein levels of phosphorylated protein corrected by total levels relative to control, time 0, AKT (upper) or GSK3β (lower). N = 3, ANOVA, ***p < 0.001, **p < 0.01, *p < 0.05. **(E–F)** HeLa control or OCRL1 silenced cells were serum starved for 4 h and then incubated with 100 nM insulin ± 50 μM YU1426670 for indicated periods of time. **(E)** Cell lysates were analyzed by western blot to detect total and phosphorylated forms of AKT and GSK3β. **(F)** Graph corresponds to the protein levels of phosphorylated protein corrected by total levels relative to control, time 0, AKT (upper) or GSK3β (lower). N = 3 ANOVA, ***p < 0.001, **p < 0.01, *p < 0.05.

Considering this insulin effect on megalin phosphorylation and cell surface expression, we were interested to know if LS cellular models respond differently to insulin (Figure 7). We measured insulin signaling up to 2 h in LLC-PK1 cells treated with 50uM of YU142670 to inhibit OCRL1. Cells showed a decreased insulin response, evaluated by a lower activation of AKT at 15 min of activation (Figures 7A,B). In order to test if the absence of OCRL1 affects insulin signaling in a different cell type, we tested Hela cells, a human cell line that does not express megalin. Hela cells were treated with YU142670 or vehicle (Figures 7C,D) or silenced for OCRL1 (Figures 7E,F). There was an evident difference in the time of response to insulin in Hela cells compared to LLC-PK1. In Hela cells, the phosphorylation peak of AKT was at 5 min of insulin stimulation. In both models (cells treated with YU142670 and OCRL KD), the response to the hormone was significantly decreased compared to the control cells, specifically by reducing AKT phosphorylation. Similar results were observed regarding pGSK3ß. Therefore, cells with decreased function of OCRL1 are less responsive to insulin. Similarly, we tested insulin response in APPL1 KD Hela cells, finding that both AKT and its downstream substrate GSK3 show a reduction in phosphorylation (Supplementary Figure S5).

Overall, these results indicate that insulin signaling operates under cellular LS conditions, although with significantly reduced efficiency. Potentially, insulin could be considered a tool to reduce the constitutive GSK3ß-mediated megalin phosphorylation, which decreases the receptor cell surface levels (Yuseff et al., 2007).

## Discussion

The elucidation of the molecular mechanisms underlying the regulation of megalin function and trafficking is a central issue in understanding the receptor’s role in human pathologies. In the present paper, we focused on the regulation of megalin by the phosphatidylinositol 5-phosphatase OCRL1 in a cellular model that simulates the LS condition specifically at the level of the proximal tubule of the kidney. First, we show evidence of megalin down-regulation protein and a reduction of the receptor shedding under the OCRL1 silencing condition reinforcing the proposed trafficking defects of megalin in LS renal phenotype (Pisitkun et al., 2004; Erdmann et al., 2007; Alexander and Grinstein, 2009; Suruda et al., 2017). Megalin proteolysis could be of physiological importance in regulating its own expression due to the transcriptional role proposed for its intracellular domain in the nucleus (Li et al., 2008; Marzolo and Farfán, 2011). The mechanisms underlying the shedding process include ligand binding, different metalloproteinases, and PKC-dependent regulation (Zou et al., 2004; Marzolo and Farfán, 2011; Mazzocchi et al., 2017). In the present study, we complement these observations by showing the importance of OCRL1 and its interacting endosomal protein APPL1 in regulating total and cell surface levels of megalin and the secretion of the receptor ectodomain. Besides, we described how OCRL1 regulates the megalin phosphorylation and the endosomal distribution of the receptor depending on its phosphorylation status. Finally, and physiologically relevant, we showed that megalin phosphorylation and cell surface expression is regulated by insulin.

In our proximal tubule cells model for the Lowe condition, endogenous megalin was reduced without changes in the receptor’s mRNA, similar to what was found in the humanized Lowe Syndrome mouse model (Festa et al., 2019). The reduction in megalin protein, observed in OCRL1 silenced LLC-PK1 cells, is similar to what was reported in a mouse model of Dent1 disease, another pathology with a related renal phenotype as LS (Christensen et al., 2003). Dent1 disease occurs due to gene mutations of the chloride channel CLC-5 (Fisher et al., 1995), a protein that interacts with megalin and regulates its trafficking in renal proximal tubule cells (Christensen et al., 2003). Moreover, the evaluation of megalin, secreted in the urine of Dent patients, also shows a reduction in the presence of the receptor (Norden et al., 2002). Thus, our results corroborate the evidence indicating a correlation between renal defects that affect megalin function in LS and Dent1 disease (Norden et al., 2002; Shrimpton et al., 2009; Vicinanza et al., 2011).

In revealing megalin degradation in cells with reduced OCRL1 protein, we found that protein stability, but not its synthesis, was decreased upon OCRL1 silencing. In the absence of OCRL1, cargoes accumulate in EEA1-positive early/sorting endosomes and cannot recycle from tubular structures to the plasma membrane (Vicinanza et al., 2011). Therefore, we initially speculated that in LLC-PK1 cells deficient in OCRL1 the receptor could be favored to get into the degradative lysosomal pathway, explaining the significant reduction in megalin protein. Our results in cells with inhibition of lysosomal activity (Supplementary Figure S1) added to the similar presence of megalin in Rab7-late endosomes in control and inhibited OCRL1 cells (Figure 5), did not support this possibility. In contrast, our data favor the role of the proteasomal degradation pathway in megalin half-life. Another possibility to explain the decrease of megalin in OCRL1 silenced LLC-PK1 cells relates to the role of the enzyme dipeptidyl-peptidase 4 (DPP4) (Aroor et al., 2016). This enzyme plays a relevant role in the proximal tubule (Lee et al., 2015). DPP4 is activated by EGFR stimulation and is associated with a reduction of megalin in mice (Aroor et al., 2016). In this regard, it is worth mentioning that in OCRL1 KD cells, the activation level of EGFR is significantly increased due to its accumulation in endosomes, where it persists in its signaling mode, activating ERK (Vicinanza et al., 2011). The total protein levels and the half-life of megalin were reduced in APPL1 silenced cells, similar to what was found in OCRL1 KD cells.

To study the role of OCRL1 and APPL1 in megalin ectodomain secretion, we first validated mMeg as a proper model to study the proteolysis (shedding) and trafficking of megalin. It has been reported that the overexpression of the expected product of *γ*-secretase activity over the MCTF, to the so-called megalin intracellular carboxy-terminal domain (MICD), down-regulates megalin and also a Na+/H+ exchanger 3 at a transcriptional level in a proximal tubule cell lines (Li et al., 2008). However, we were unable to detect this proteolytic product in our system. The expected MICD has been challenging to identify at the moment in tissue and cell cultures. Using inhibitors of proteasomal degradation, we have tried unsuccessfully to detect MICD (data not shown). Despite these results, our cellular models allow us to establish a role of OCRL1 as well as APPL1 in megalin regulation; the silencing of these proteins reduced both cell surface levels and megalin secretion to the extracellular media (corrected by the total levels of the receptor), compared to the control cells in kidney epithelial cells, LLC-PK1 and MDCK. The reduction of megalin cell surface levels in OCRL1 knock-down is also explained by an impairment of the receptor recycling from the sorting endosome, as was already described (Vicinanza et al., 2011).

Among the possible mechanisms underlying the reduction in megalin shedding observed in OCRL1 and APPL1 knock-down cells is the reduced presence of megalin at the cell surface. In general, the events triggered by MMPs and ADAM proteins metalloproteinases occur at the plasma membrane (Edwards et al., 2008; Larios and Marzolo, 2012; Zakiyanov et al., 2019). In lung epithelial cells, the metalloproteinases directly associated with megalin proteolytic processing are MMP-2 and MMP-14 (Mazzocchi et al., 2017) (also known as MT1-MMP). MMP-2 is secreted as a pro-enzyme, activated by other metalloproteinases, including MMP-14, and binds to the megalin ectodomain (Mazzocchi et al., 2017). MMP-14 directly interacts with and processes megalin (Mazzocchi et al., 2017) and is also present in the proximal tubule (Zakiyanov et al., 2019). Like megalin, MMP-14 localizes at the apical surface and is found in apical vesicles in epithelial cells from the prostate and uterus (Thathiah and Carson, 2004; Sroka et al., 2008). Besides, MMP-14 can be released in exosomes in an active form (Hakulinen et al., 2008) and potentially could process exosomal megalin present in the urine (Marzolo and Farfán, 2011; De et al., 2017). Besides the reduction of surface megalin, it is also conceivable that the trafficking of these metalloproteinases could be affected in the LS condition, either its presence at the cell surface or its intracellular activity. MMP-14 traffics through the endosomal pathway and in this could be affected by an OCRL1 deficiency; MMP14 is endocytosed and localized first to early endosomes and later gets recycled through either early endosomes and the trans-Golgi network (TGN) (Wang et al., 2004) or late endosomes (Hakulinen et al., 2008; Williams and Coppolino, 2011; Macpherson et al., 2014). Megalin could also be a substrate of the ADAMs family (Zou et al., 2004; Larios and Marzolo, 2012). These membrane proteins locate in perinuclear regions, but they are also found in lesser amounts at the cell surface (Edwards et al., 2008), where they process many of their substrates, such as adhesion proteins and surface receptors (Reiss et al., 2006; Murphy, 2009). However, active forms of ADAMs are also found in intracellular compartments (Shirakabe et al., 2001); for example, ADAM10 and 17/TACE induce the shedding of APP in the TGN (Skovronsky et al., 2000). In the same direction, ADAM10 efficiently processes CD23 and possibly other substrates at the endosome (Mathews et al., 2010), and therefore, its activity could be directly affected in an LS condition.

The similar results obtained upon APPL1 silencing are probably due to the role that this adaptor protein has in the recruitment of OCRL1 to early endosomes (McCrea et al., 2008; Noakes et al., 2011; Bohdanowicz et al., 2012), besides the functions of the small endosomal GTPases Rab5 and Rab35 have in the recruitment of the phosphatase (Hyvola et al., 2006; Kanno et al., 2008; Dambournet et al., 2011). On the other hand, the internalization pathway of megalin includes its trafficking through APPL1 positive early endosomes; however, in cells lacking OCRL1, the presence of internalized megalin in APPL1 endosomes is reduced, contrasting with its increase in early endosomes positive for EEA1 (Vicinanza et al., 2011; Festa et al., 2019). These observations imply that even when megalin interacts with APPL1 (Erdmann et al., 2007), its presence in endosomes positive for this adaptor protein requires the presence of OCRL1.

Considering the negative role that the GSK3ß-mediated phosphorylation of the cytoplasmic domain of megalin has in receptor surface expression (Yuseff et al., 2007), we evaluated this modification in our cellular models. Our first hypothesis was that a defect in the phosphorylation of megalin, reflected by higher levels of this modification, could explain the lower surface levels of megalin in OCRL1 silenced cells. Cells with decreased levels of OCRL1 showed no change in basal GSK3ß activity, but, in contrast to our prediction, they exhibited lower levels of megalin phosphorylation. There is no information concerning where GSK3ß phosphorylates megalin and how this phosphorylation is controlled by the action of phosphatases or other kinases. However, it is known that AKT controls GSK3ß activity when both proteins are in PI (3,4) P2-enriched endosomal membranes and recruited by APPL1 (Schenck et al., 2008) suggesting that megalin phosphorylation would occur in the endosomal pathway. Then, we hypothesized that inhibition of OCRL1 could decrease the colocalization of GSK3ß with megalin. However, our results did not support this option, at least using our experimental strategy. On the other hand, megalin is also phosphorylated by PKC, PKA and CKII, although to a much smaller extent than by GSK3ß (Yuseff et al., 2007), but the physiological significance of these modifications is still unknown. We could speculate that under LS conditions, the non-GSK3ß dependent phosphorylation of megalin and mediated by any of the kinases mentioned, could be inhibited explaining part of the reduction in megalin phosphorylation observed in OCRL KD cells. If any of these phosphorylation sites would have a positive role in the expression of megalin at the plasma membrane, their inhibition could result in a reduction of cell surface megalin. This effect would add to the recycling impairment caused by the accumulation of actin cytoskeleton at the endosomal membrane (Vicinanza et al., 2011). Future experiments would be required to address this possibility*.*

Of note, there was residual but still significant phosphorylation of megalin in our LS cellular model susceptible to inhibition of GSK3ß with LiCl. Considering this finding, we tested a physiological stimulus that activates PI3K and AKT as insulin, to reduce GSK3ß activity and megalin phosphorylation. For the first time, our results showed that insulin significantly reduced megalin phosphorylation and increases its expression at the plasma membrane under normal conditions. Moreover, in LLC-PK1 cells with reduced levels of OCRL1 and APPL1, the treatment with insulin significantly decreased GSK3ß activity and megalin phosphorylation, suggesting that this signaling pathway is still functional under these conditions. Accordingly, insulin treatment also increased megalin cell surface expression in cells with reduced OCRL1 activity. Potentially, the insulin pathway could be considered a tool to reduce the constitutive GSK3ß-mediated megalin phosphorylation and increase the receptor cell surface levels (Yuseff et al., 2007).

LS patients exhibit a urinary waste of several growth factors, including insulin (Norden et al., 2001; Bökenkamp and Ludwig, 2016). From this point of view, it is expected that insulin signaling could be affected in LS as we found in our study. Moreover, the reduction in insulin signaling evidenced in our experiments at the cellular level, specifically in conditions of reduction of OCRL1 function, not only would reduce megalin recycling but also would potentially decrease megalin endocytosis as pAKT is required for the efficient megalin-mediated endocytosis of albumin, a physiologically relevant ligand of the receptor present in the proximal tubule (Silva-Aguiar et al., 2022). Regarding AKT activity, it has been recently found that the mTORC1 complex is inactivated in OCRL1 deficient cells (Madhivanan et al., 2020), a defect that triggers a lack of nutrient-sensing (Wang et al., 2021) due to mTORC1 is required for proper insulin signaling (Saltiel and Kahn, 2001). Insulin signaling is also highly dependent on the cell type, something we observed in our experiments, and is associated with insulin receptor trafficking (Iraburu et al., 2021). For instance, the activation of PI3K and pAKT takes place at the plasma membrane as well as in endosomes, with higher activation at the endosome (Ceresa et al., 1998; Iraburu et al., 2021), being possible that under LS conditions at least the endosomal signaling, would be affected. Therefore, it is expected that the response to insulin and megalin phosphorylation mediated by GSK3ß are affected in LS cellular models and, in general, cells with decreased function of OCRL1 would be less responsive to insulin. Moreover, in LS patients’ insulin signaling could be partially inhibited, affecting other processes including the stimulation of megalin expression under chronic kidney disease (Hosojima et al., 2009; Bryniarski et al., 2018) and the insulin-mediated surface expression of GLUT4, a trafficking response that is inhibited by GSK3ß (Duan X et al., 2021).

Endosomal compartments are part of the trafficking route of cargo proteins. They also have roles in membrane turnover and intracellular communication being a platform characterized by endosomal adaptor proteins, where components of signaling pathways are recruited (Vicinanza et al., 2011). Regarding insulin, APPL1 has been identified as an AKT-interacting endosomal adaptor (Mitsuuchi et al., 1999; Schenck et al., 2008) required for insulin signaling (Sato et al., 2007; Ryu et al., 2014). Here, we present insulin signaling kinetics showing a similar disruption of AKT and GSK3ß phosphorylation upon APPL1 KD and OCRL1 KD cells.

As mentioned before, in LS and Dent disease there are significant decreases in A-megalin in the urine (Suruda et al., 2017). Furthermore, other pathologies leading to chronic kidney diseases, such as diabetes (Ogasawara et al., 2012; De et al., 2017) and IgA nephropathy (Seki et al., 2014), exhibit an increase in the presence of C-megalin, probably secreted into the urine as exosomes. With the evidence described in this work, we highlight the importance of establishing cellular models for studying proteolysis, trafficking and phosphorylation of megalin in pathologies in which this receptor is affected to find potential therapeutic tools for these diseases.

## Data Availability

The original contributions presented in the study are included in the article/Supplementary Material, further inquiries can be directed to the corresponding author.
