# Supplementary material for: Participation of OCRL1, and APPL1, in the expression, proteolysis, phosphorylation and endosomal trafficking of megalin: Implications for Lowe Syndrome
Source: Front Cell Dev Biol. 2022 Oct 20;10:911664. doi: 10.3389/fcell.2022.911664 (PMC9630597; doi:10.3389/fcell.2022.911664)
Supplement: Supplementary file 1 [file DataSheet2.pdf]

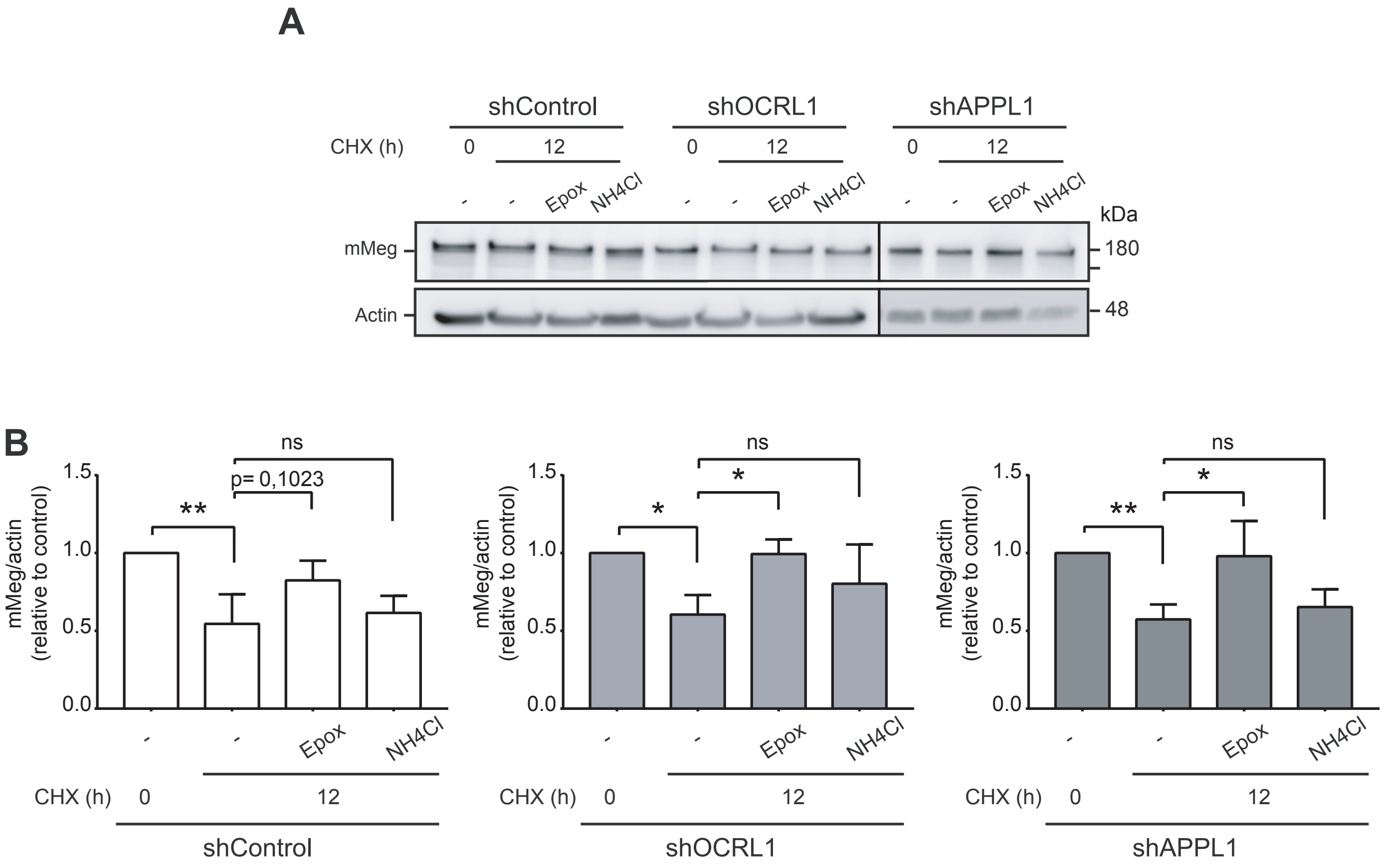

Supplementary Figure 1. mMeg degradation under proteasomal and lysosomal inhibition. (**A**) mMeg-LLC-PK1 silenced cells were treated or not with cycloheximide (CHX; 100 µM) for 12 h. Cells incubated with CHX were co-incubated with 1 µM of Epoxomicin (EPOX), 25 mM NH_4Cl or vehicle followed by the determination of mMeg and actin protein levels by western blot. (**B**) Quantification of mMeg protein levels corrected with actin as load control. Data are expressed as the means ± SEM of four independent experiments. ANOVA, *P < 0.05, **P < 0.01, ns = no significance

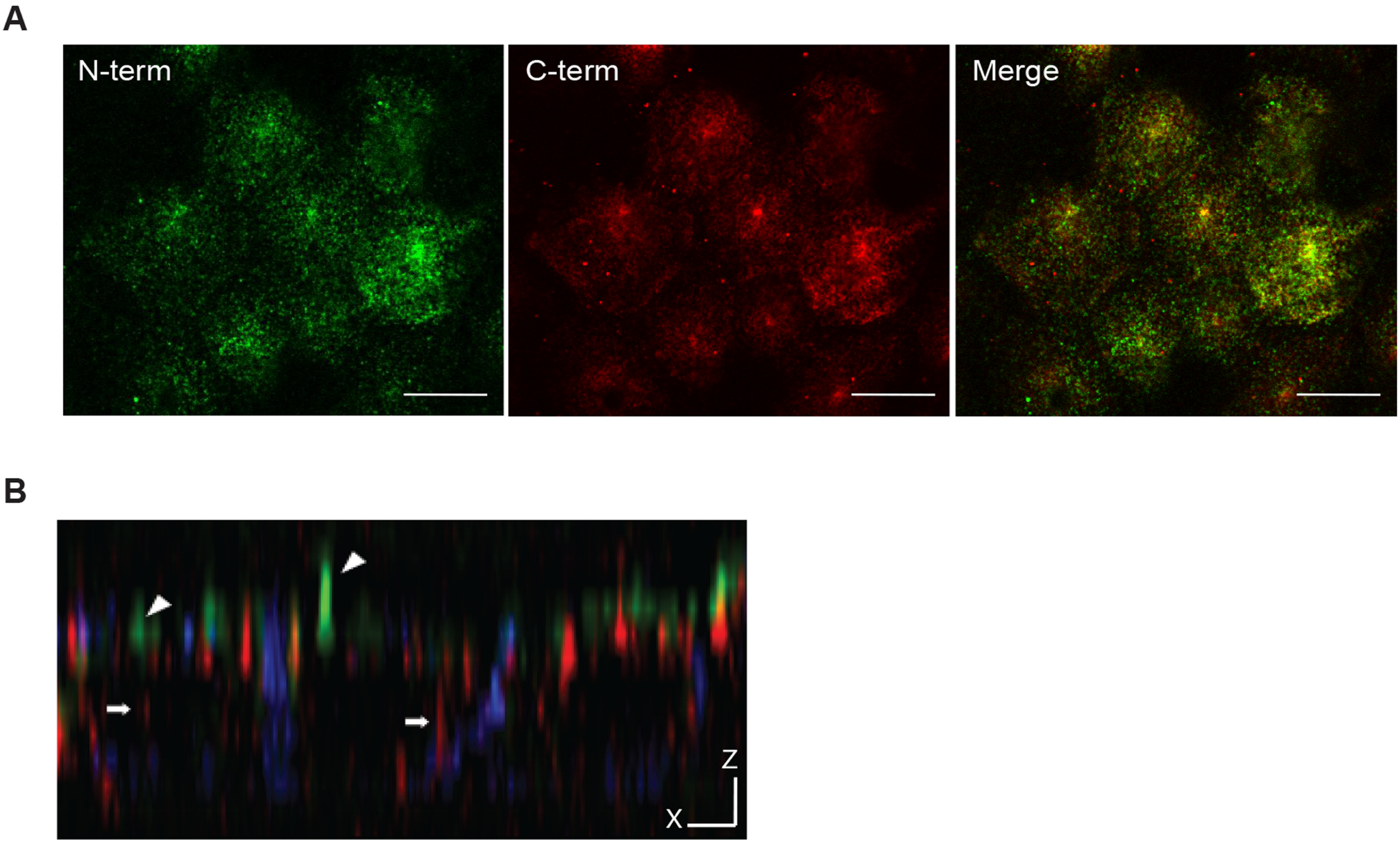

Supplementary Figure 2. Detection of megalin fragments in polarized mMeg-MDCK cells. Cells were grown on Transwell filters until they were polarized. (**A**) The detection of amino-terminal (N-terminal) or carboxy-terminal (C-terminal) domains of mMeg was performed by immunofluorescence with anti-HA (green) and anti-megalin (red) primary antibodies, respectively. The image was acquired by confocal microscopy (Z-stack), the image shows an apical view in XY plane. (**B**) Image of X-Z plane of the image shown in A. Additionally, the basolateral marker (E-cadherin, blue) was detected. Arrows show megalin proteolytic products containing the intracellular domain of the receptor and arrowheads show megalin ectodomain.

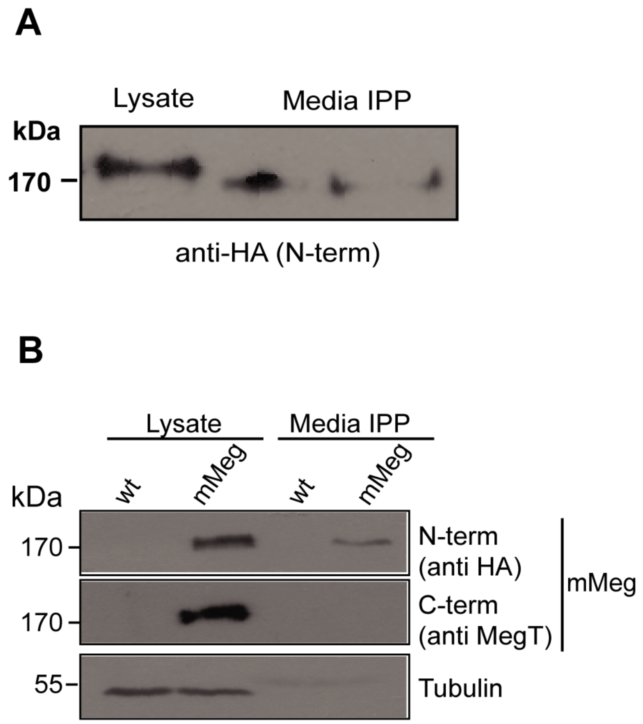

Supplementary Figure 3. Proteolytic product of megalin is secreted to the culture media. (**A**) mMeg-MDCK cells were grown to confluence for 48 h. The conditioned medium was concentrated and then immunoprecipitated with an anti-HA antibody. mMeg and its N-terminal fragments were detected by western blot with an anti-HA antibody from the immune complex and the cell lysates. (**B**) Western blot detecting amino (anti-HA) and carboxy-terminal (anti-MegT) fragments of mMeg immunoprecipitated from the media and lysate from wild-type and mMeg-MDCK cells. Tubulin was used as a loading control.

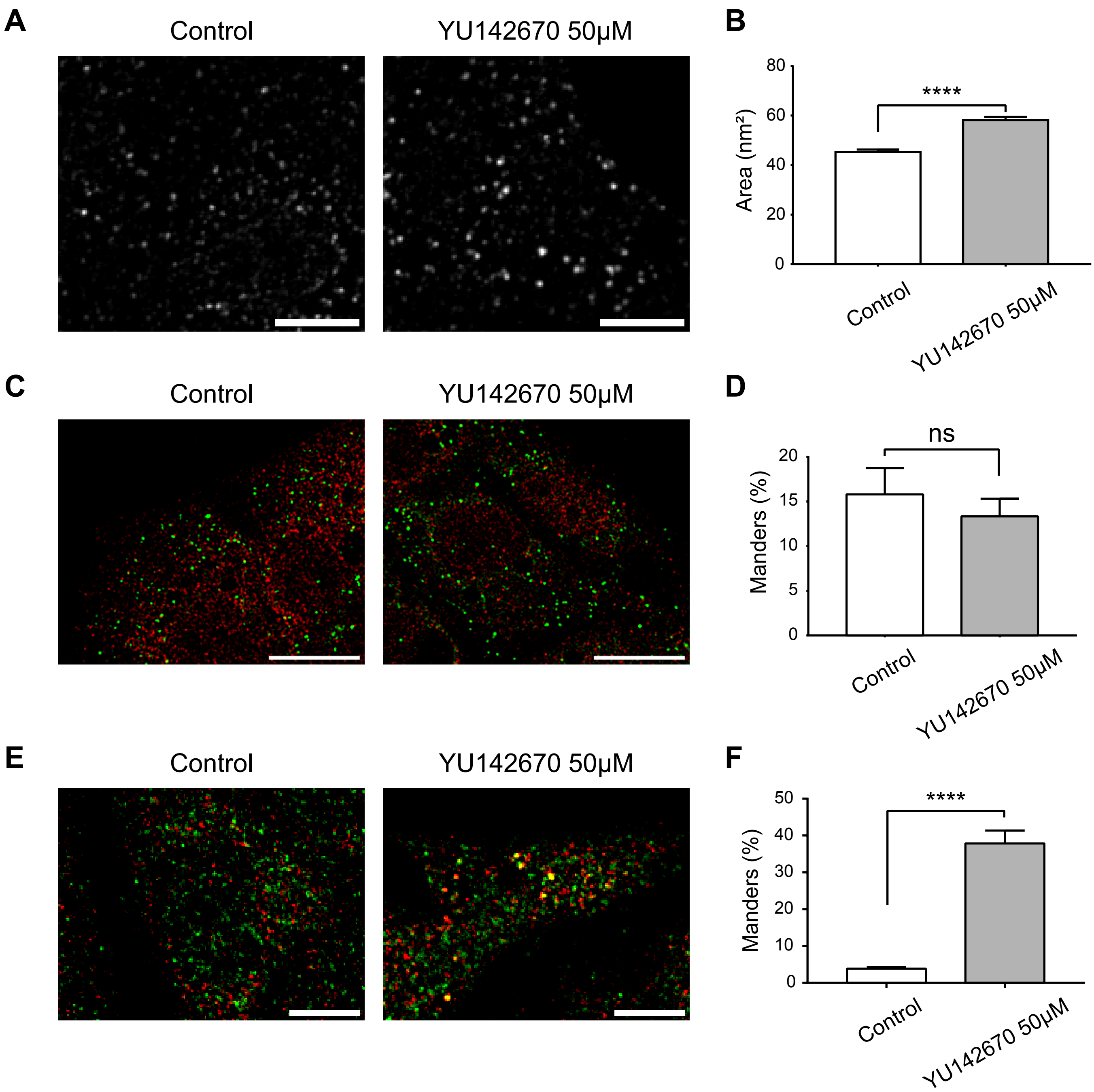

Supplementary Figure 4. OCRL1 inhibitor (YU142670) increases Early Endosomes size without changing megalin colocalization with GSK3β. (**A**) LLC-PK1 cells were treated with 50µM YU142670 or vehicle for 4 h. Cells were processed for fluorescence microscopy and stained with anti-EEA1 antibody. Shown are representative confocal images. Scale Bar 10 µm. (**B**) Images were analyzed with Image J to measure the endosomal area of EEA1 staining. At least 60 cells were analysed. Mann Whitney test, ****$P < 0.0001$. (**C**) LLC-PK1 cells were transfected with HA-tagged GSK3β. After 24 h of expression, the cells were treated with 50µM YU142670 or vehicle for 4 h, fixed and stained with anti-HA (red) and anti-megalin (green) antibodies. Scale Bar 20 µm. (**D**) Images were analyzed with Image J to measure the Manders percentage between HA and megalin structures. At least 35 cells were analysed. Mann Whitney test, ns = no significance. (**E**) LLC-PK1 cells were transfected with mCherry-Rab11, treated with 50µM YU142670 or vehicle for 4 h, fixed, and stained with anti-EEA1 (green). Scale Bar 10 µm. (**F**) At least 50 cells were analyzed with Image J to measure the Manders percentage between EEA1 endosomes and mCherry-Rab11 structures. ANOVA, ****$P < 0.0001$.

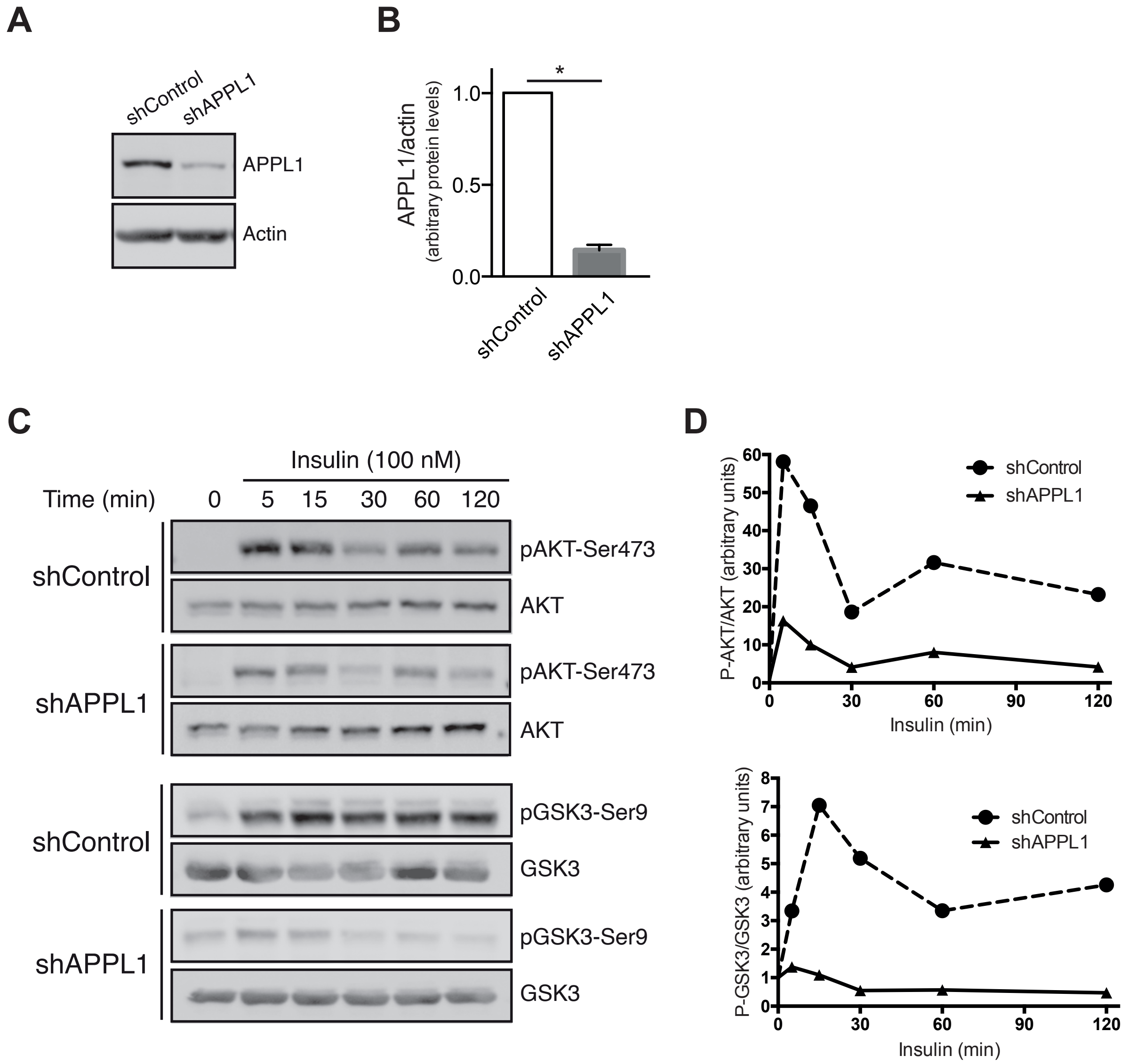

Supplementary Figure 5. Inhibition of insulin signaling in APPL1 silenced HeLa cells. (**A**) Protein levels of APPL1 and actin were analyzed in whole-cell lysates of control and APPL1 silenced HeLa cells by western blot. (**B**) Quantification of APPL1 levels corrected with actin levels in control and APPL1 KD cells. Data are expressed as the means ± SEM of three independent experiments (t-test, **P < 0.01). (**C**) Control and APPL1 silenced HeLa cells were serum starved for 4h and then incubated with 100nM insulin for indicated periods of time. Cell lysates were analyzed by western blot to detect GSK3ß and AKT, total and phosphorylated forms. (**D**) Graph corresponds to the protein level (insulin signaling in B) of phosphorylated protein corrected by total levels, AKT (upper) or GSK3ß (lower).
