# Supplementary material for: Participation of OCRL1, and APPL1, in the expression, proteolysis, phosphorylation and endosomal trafficking of megalin: Implications for Lowe Syndrome
Source: Front Cell Dev Biol. 2022 Oct 20;10:911664. doi: 10.3389/fcell.2022.911664 (PMC9630597; doi:10.3389/fcell.2022.911664)
Supplement: Supplementary file 2 [file DataSheet1.PDF]

Supplementary Methods

Antibodies and Reagents

Cycloheximide (CHX; C7698-1) was provided by Sigma (St. Louis, MO). Epoxomicin (EPOX; 10007806) was supplied by Cayman (Ann Arbor, Michigan). Ammonium chloride (NH_4Cl; A649-500) was purchased from Fisher Scientific (Hampton, New Hampshire, USA). Tissue culture-treated Transwell polycarbonate filters were from Costar (Cambridge, MA, USA). GSK3-HA plasmid was previously described (Marzolo and Farfán, 2011).

Megalin degradation assays

To determine mMeg levels under proteasomal or lysosomal inhibitors, control, OCRL1 silenced or APPL1 silenced cells were co-treated with 100 µM CHX plus 1 µM EPOX or 25 mM NH_4Cl for 12 h. Cells were lysed and the presence of megalin and actin were visualized by western blot.

Megalin detection in polarized MDCK cells grown on filters

mMeg-MDCK cells were grown to confluence in Transwell, fixed with 4 % paraformaldehyde in PBSc (with 1mM calcium) and permeabilized with 0.2% Triton X-100 in PBSc. Next, the cells were blocked with 0.2% gelatin in PBSc and incubated successively with the primary antibodies (anti-HA, anti-megalin, anti-E-cadherin) and the corresponding secondaries. The mouse monoclonal anti-HA (Marzolo et al., 2003) detected the N-terminal portion of mMeg and the rabbit polyclonal anti-megalin (Marzolo et al., 2003) to recognize the C-terminal domain of the receptor. Samples were washed with PBS and incubated with secondary antibodies Alexa488 goat anti-mouse IgG and Alexa594 goat anti-rabbit IgG. Images were captured with Zeiss Axiovert 200M and LSM 5 Pascal laser scanning confocal microscope and processed with ImageJ software (NIH).

Detection of proteolytic product of megalin in culture media.

mMeg expressing MDCK cells were grown to confluence for 48 h. The conditioned medium was collected, concentrated and immunoprecipitated with an anti-HA antibody. Samples were boiled in Laemmli sample buffer (62.5 mM Tris-HCl, pH 6.8; 2% w/v SDS, 10% v/v glycerol and 5% ß-mercaptoethanol) and separated by SDS-PAGE. mMeg and its N-terminal fragments were detected by western blot with an anti-HA antibody and the carboxy-terminal fragments were detected with rabbit polyclonal anti-megalin (Marzolo et al., 2003)

Insulin Signaling

Control or shAPPL1 cells, were serum-starved for 4 h. Then, cells were incubated with 100nM insulin for different times, to promote AKT and GSK3β phosphorylation due to insulin signaling. Cells were washed twice with PBS, lysed, and analyzed by western blot.

Statistical Analysis

The blots were quantified with the ImageJ software. Data were expressed as the means ± SEM from at least three independent experiments. Comparisons of two conditions were performed using Mann-Whitney's test. For multiple comparisons data was analyzed using ANOVA with Bonferroni correction. The statistical analyses and graphs were performed using GraphPad Prism 5.
